# Numerical algebraic geometry for model selection and its application to the life sciences

**DOI:** 10.1098/rsif.2016.0256

**Published:** 2016-10

**Authors:** Elizabeth Gross, Brent Davis, Kenneth L. Ho, Daniel J. Bates, Heather A. Harrington

**Affiliations:** 1 Department of Mathematics, San José State University, San José, CA 95112, USA; 2 Department of Mathematics, Colorado State University, Fort Collins, CO 80523, USA; 3 Department of Mathematics, Stanford University, Stanford, CA 94305, USA; 4 Mathematical Institute, University of Oxford, Oxford OX2 6GG, UK

**Keywords:** model validation, polynomial optimization, maximum-likelihood, chemical reaction networks, parameter estimation

## Abstract

Researchers working with mathematical models are often confronted by the related problems of parameter estimation, model validation and model selection. These are all optimization problems, well known to be challenging due to nonlinearity, non-convexity and multiple local optima. Furthermore, the challenges are compounded when only partial data are available. Here, we consider polynomial models (e.g. mass-action chemical reaction networks at steady state) and describe a framework for their analysis based on optimization using numerical algebraic geometry. Specifically, we use probability-one polynomial homotopy continuation methods to compute all critical points of the objective function, then filter to recover the global optima. Our approach exploits the geometrical structures relating models and data, and we demonstrate its utility on examples from cell signalling, synthetic biology and epidemiology.

## 1. Introduction

Across the physical, biological and social sciences, mathematical models are formulated and studied to better understand real-world phenomena. Often, multiple models are developed to explore alternative hypotheses. It then becomes necessary to choose between different models, for example, based on their fit with noisy experimental data. This is the problem of model selection, a fundamental scientific problem with practical implications [1–3].

When dealing with deterministic models in the life sciences, the standard approach to model selection is to first estimate all model parameters and hidden variables from the data, then select the model with the smallest best-fit error, up to some penalty on model complexity [4,5]. Thus, at its core, model selection is intimately tied to parameter estimation. For example, if we have a model described by a system of equations *f*(*a*, *x*) = 0 in the parameters *a* and variables *x* with measurable ‘outputs’ *z* = *g*(*x*), then parameter estimation can be written as the following least-squares optimization problem:1.1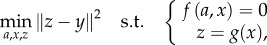;where *y* denotes the observed data, i.e. measured outputs. Unless *f* and *g* are convex, solving (1.1) is a non-convex problem, which can be challenging as standard local solvers run the risk of getting trapped in local minima (especially in high dimensions). This can be mitigated somewhat with techniques such as simulated annealing [6,7] or convex relaxation that has been successful for model invalidation [8–10], but there is generally no guarantee that a global minimum will be found.

When *f* and *g* are polynomial, however, problem (1.1) can be solved globally by finding all roots of an associated polynomial system. In this case, ideas from computational algebra and algebraic geometry can be effective; see, e.g. [11–14] for applications of Gröbner bases in systems biology and [15] for applications of algebraic geometry to statistical inference. Such symbolic methods tend to be computationally expensive, which limits their use in practice and are bypassed here. Thus, although they provide a solution in principle, new algorithms and techniques are yet desired.

In this paper, we aim to fill this gap by proposing a framework for global parameter estimation for polynomial deterministic models using numerical algebraic geometry (NAG), a suite of tools for numerically approximating the solution sets of multivariate polynomial systems via adaptive multi-precision, probability-one polynomial homotopy continuation [16,17]. This is a deterministic method, so it will produce the same results (up to numerical error) every time. Unlike other approaches, there is no sense of ‘simulations' or sampling required for this method. Our approach scales well in dimension relative to classical symbolic methods [18], and, while it comes with a higher computational cost than standard local optimization, it has a probability-one guarantee to recover the global optima, solving problem (1.1) in the strong sense. This allows us to reason rigorously about model fit and to address the related problems of model selection and parameter estimation from a maximum-likelihood perspective. We demonstrate our techniques on examples from biology, where polynomial models often arise as the steady-state descriptions of mass-action chemical reaction networks. Although some limitations remain, we believe that this work achieves its primary purpose of introducing NAG as a valuable complement to existing tools for model evaluation and analysis. Additionally, this paper highlights specific challenges that arise when using polynomial methods for model inference, such as dealing with positivity constraints and non-isolated solutions, and provides guidance for tackling these challenges.

The remainder of the paper is organized as follows. In §2, we state precisely the problems with which we are concerned: model validation, model selection, and parameter and hidden variable estimation. We then present the NAG algorithms for solving each problem. Finally, we showcase our approach on a few examples, including cell death activation, synthetic biocircuits, human immunodeficiency virus (HIV) progression and protein modification.

## 2. Problem statement

Consider a model whose dynamics are described by the system of polynomial differential equations2.1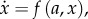;where 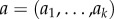 are parameters (e.g. rate constants in a deterministic mechanistic model, such as a chemical reaction network with mass-action kinetics), 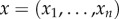 are variables, and 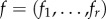 are polynomials in *x* and *a* with measurable outputs 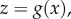 where 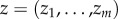, *m* ≤ *n* and 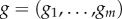 are polynomials in *x*. While the parameters *a*_1_, … ,*a_k_* are treated as fixed variables in our exposition, we separate them from *x*_1_, … ,*x_n_* to respect how such variables are treated differently in experimental and computational settings.

In algebraic geometry, a *variety* is a solution set of a system of polynomial equations; we use this terminology for our next two definitions. The *real model variety* is the solution set of the system2.2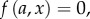;2.3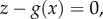;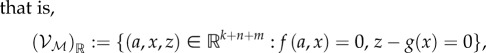;

corresponding to the steady states of the model. Now, consider, for simplicity, the case of a single data point 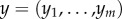. (See the electronic supplementary material for multiple data points.) The *real data variety* is then the affine linear space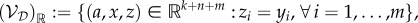;with 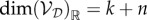. We consider the case when the data include some extrinsic (measurement) noise; we assume the errors 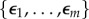 on the observed data variables are uncorrelated random variables and each error 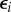 is normally distributed with known variance *σ_i_* (which can be obtained by instrument calibration).

Using this geometric framework, the problems of (1) model validation, (2) model selection and (3) parameter estimation can be described precisely in terms of the real algebraic varieties 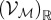 and 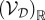.

### 2.1. Problem 1: model validation

For model validation, we want to determine whether a deterministic polynomial model 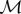 is compatible with the data according to a given significance level *α*. Using the noise assumptions from above, each deterministic model 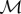 gives rise to an associated statistical model. Specifically, given a deterministic system 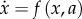 with an observation *y* made at steady state, the statistical model under consideration is2.4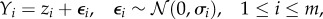;2.5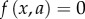;2.6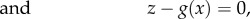;where *x*, *a*, *z* are all unknown, and *σ_i_* is known for all *i*.

The compatibility question is akin to significance testing and asking whether the model is a ‘good fit’ for the data. A natural goodness-of-fit statistic is2.7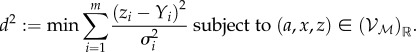;When the variances differ, *d*^2^ is the minimum-squared weighted Euclidean distance between 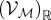 and 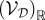. When all variances are equal to one, the value of (2.7) is just the minimum-squared distance. For the remainder of the text, we assume the latter, knowing that we can simply rescale.

Optimization problem (2.7) can be derived directly from the log-likelihood function2.8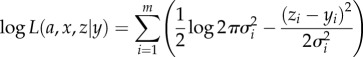;as demonstrated in §2.1 of the electronic supplementary material. Indeed, minimizing the argument of (2.7) over 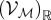 is equivalent to maximizing 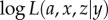 over 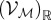. This provides a bridge to standard statistical model selection tools such as AIC [19] and BIC [20]. Note that *a* and *x* do not appear in (2.8), instead, they appear in the constraints of the problem.

In model validation, our null hypothesis is that the data *y* are generated from the statistical version of the deterministic model 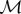. If the data are generated from the model 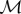 with normally distributed extrinsic noise, then the distribution function of *d*^2^ is dominated by that of the chi-squared distribution with *m* degrees of freedom, 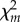, where *m* is the number of measurable outputs. Thus, if *p_α_* is the upper α-percentile for 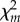, then 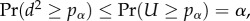 where 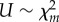. We reject the model 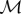 as *incompatible*, i.e. we reject the null hypothesis, if the observed value of *d*^2^ is greater than *p_α_*; otherwise, we say that the model 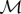 is *compatible*.

If the real model and data varieties intersect, that is, 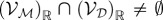, then *d*^2^ = 0, and we also say that the model is compatible with the data. If there are restrictions on (*a*,*x*,*y*), for example, if all parameters and variables are required to be non-negative, then finding *d*^2^ becomes a *constrained optimization* problem (see electronic supplementary material).

### 2.2. Problem 2: model selection

For model selection, we are given a set of models 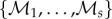 and want to determine the model of best fit. In this setting, we use the statistic *d*^2^ to make a selection, either by choosing the model with the smallest value of *d*^2^ or by using *d*^2^ in conjunction with a complexity penalty, similar to the Bayesian or Akaike information criteria [1].

If the statistic *d*^2^ evaluates to zero for all (or even multiple) models, then we are unable to make a selection between the models. This can be remedied by designing experiments that yield more informative data. For example, measuring more variables can reduce the dimension of 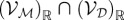; the most informative situation is when 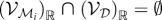 for all models. This indeterminacy can also be resolved by taking multiple measurements and minimizing the joint squared distance (see the electronic supplementary material).

### 2.3. Problem 3: parameter estimation

Parameter estimation can be achieved by finding the point 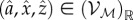 that minimizes the value of (2.7). The point 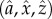 is the maximum-likelihood estimate under the given noise assumptions. The parameter estimate is then 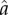; the estimate of the hidden variables, 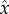 and the estimate of the ‘de-noised’ outputs, 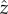. Of course, if the data and model varieties intersect, then there will be one or more (possibly infinite) choices for 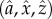. Otherwise, it is a matter of solving a polynomial system that yields the point(s) on 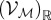 nearest 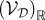. This is described in more detail in §3.

## 3. Geometry

In each of the problems stated above, we seek to minimize the distance between 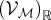 and 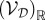 or to find the intersection of these two sets. Standard methods for solving nonlinear optimization problems are local in nature, i.e. only guaranteed to converge to a local minimum which may or may not be the global minimum.

However, using NAG, we find *all* local extrema over 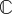, necessarily including the global minimum. Owing to this global benefit, NAG has been used before in statistical inference in the field of algebraic statistics [21,22].

Let 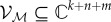 be the (complex) Zariski closure of 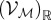 and 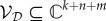 be the (complex) Zariski closure of 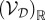. We refer to 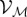 and 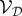 as the *model variety* and *data variety*, respectively.

The problem of determining the intersection of 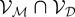 is simply a matter of solving the polynomial system obtained by taking the union of the polynomials defining 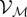 and the polynomials defining 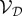. This is handled directly by NAG. If the intersection is nonempty and positive-dimensional (complex curves, surfaces, etc.), then real points can be found using the polynomial homotopy method described in [23], a method based on symbolic algorithms in [24] and, more classically, on the decision method in [25].

In the case that 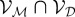 is empty, the problem of finding the points on the varieties nearest one another can also be stated in terms of a polynomial system, on which we then call NAG solvers to find solutions. A well-known necessary condition for local extrema is given by the Fritz John conditions, related to Lagrange multipliers. In the main text, we assume that 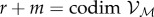; however, when this is not the case, the number of equations can be reduced (see electronic supplementary material).

**Proposition 3.1. (Equations given by Fritz John conditions)**

*Let r + m = codim* 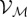
*. Let* 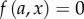
*,* 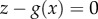 *be defined on a Zariski open set of* 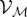
*, and define* 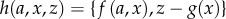 *for simplicity of notation below. If* 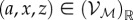 *is a local minimum of*3.1
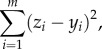
;*then there exists* 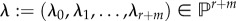, *such that* 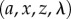 *is a solution to the system*3.2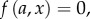;3.3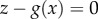;3.4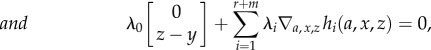;*where* 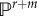 *refers to projective space, a slight generalization of complex affine space, and* 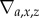 *refers to the vector consisting of all first-order derivatives with respect to all a, x, and z*.

Solving this system with NAG will provide us with all local extrema of (3.1) over 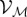, from which we may easily select the pair of nearest points. In fact, in the examples below, we use minor variations on this theme for computational efficiency; see the corresponding sections of the electronic supplementary material for details.

The geometry of zero sets of systems such as (3.2)–(3.4) is described in [26]. In particular, Draisma *et al*. [26] explore the *Euclidean distance degree* (ED degree) of a variety, which is the number of critical points of the squared distance to a general point outside the variety. The number of complex solutions to (3.2)–(3.4) when *y* is generic is the ED degree of a projection of the model variety, along the data subspace. The ED degree captures the complexity of any method that solves a squared distance minimization problem *exactly* and plays a key role in the methods discussed here. For example, the ED degree is the number of paths that need to be tracked when using the parameter homotopies described in §3.1.

### 3.1. Numerical algebraic geometry

Given a polynomial system *F* consisting of *r* polynomials and *N* variables, NAG packages, such as Bertini [16], PHCpack [27], HOM4PS-2.0 [28], use polynomial homotopy continuation to provide *probability-one* numerical methods for finding approximations of all isolated complex solutions of *F* = 0 (points) as well as *witness points* on all positive-dimensional irreducible components of the solution set of *F* = 0. These methods are probability-one in that the computations include some randomization, and this randomization will yield theoretically correct results so long as the random numbers are not chosen from some measure zero set in the parameter spaces of potential choices [17,29].

If 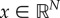 is a *real* solution of *F* = 0, it is either isolated among the complex solutions or it lies on a positive-dimensional complex irreducible component. In the former case, the methods of NAG will find *x* and recognize it as real. In the latter case, *x* can be difficult to uncover.

However, for our purposes, we usually only need to verify the existence of a real solution. In this case, we can find witness points on all positive-dimensional components and then use the procedure in §2.1 of [23] to verify the existence of real points.

Finally, there is a setting in which a particular method from NAG is especially powerful. If a parametrized polynomial system needs to be solved multiple times for varying parameter values, the *parameter homotopy* [17,29–31] can greatly reduce the computation time. Refer to the electronic supplementary material for more details, particularly §§4.1 and 4.4.

### 3.2. Algorithms

We present three related algorithms to address model validation, model selection and parameter estimation.

The aim of the first algorithm, algorithm 1, is to find the pair of points that minimize the distance between 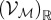 and 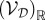. If 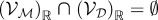, then this is obtained by solving (3.2)–(3.4); otherwise, additional techniques are required.

We also note simply that these techniques are indeed probability-one algorithms. The computations will necessarily be carried out in finite time and the steps proceed linearly (no loops), so the methods will necessarily finish.

#### 3.2.1 Algorithm 1: model validation

The computation of the intersection 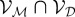 in steps 1 and 2 (table 1) can be determined in several ways. The simplest way is by considering dimensions: if 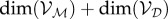 exceeds the ambient dimension, then they will almost surely intersect. If the ambient dimension is larger than the sum of the variety dimensions, then they typically do not intersect. To compute the intersection (or check to see if it is nonempty), one could substitute *y* − *g*(*x*) for (3.3) in the system (3.2)–(3.3).

**Table 1.** Algorithm 1: model validation.

input	model 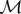 , data 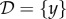 , tolerance *α*
output	*yes* or *no*
1	If 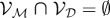 , go to step 3.
2	If 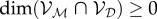 and 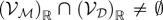 , return *yes*,
	else, go to step 3.
3	Find a pair 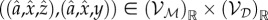 that minimizes (3.1) (using NAG software such as Bertini or PHCpack).
4	If 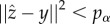 , return *yes*; else *no*.

In step 2, if 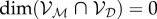, then the intersection of the two varieties consists of finitely many points; the condition 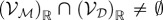 indicates that at least one of the points is real, which is straightforward to determine. If 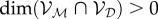, to check if 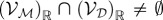, one needs more sophisticated methods, such as those in [23]. In particular, such methods will return a point in the intersection, if such a real point exists; if the point is smooth on 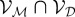, then we can also conclude that the dimension of 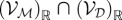 is equal to the dimension of 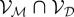.

To find the pair 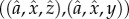 in step 3, one may solve the polynomial system (3.2)–(3.4). If there is a positive-dimensional set of (complex) extremal points, then the procedures in [23] could be used to determine if the set contains a real point.

If there are constraints on the variables or parameters, for example, if we seek to minimize (3.1) over the non-negative part of 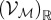, then the algorithm is updated as follows: if the 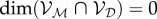 or 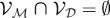, then the Fritz John equations as described in §3.1 of the electronic supplementary material should be used, while if 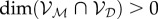, or if there is a positive-dimensional set of extremal points at step 3, then the algorithm should return *possibly*. There are methods [32–34] for finding real points, curves and surfaces within complex components of dimension 2 or less, but little is known about higher dimensions.

#### 3.2.2. Algorithm 2: model selection

In this case, there are several competing models, each with its own polynomial system. The algorithm proceeds much as in algorithm 1, but iterated for the several models under consideration. If a threshold *α* is provided, then one should first reject models that do not adequately support the data (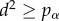), then choose the model yielding the minimum value of *d*^2^ (up to some complexity penalty). Various conclusions may be drawn, e.g. *no model adequately fits the data* or *three models adequately fit the data and model* 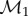 *provides the best fit*.

#### 3.2.3. Algorithm 3: parameter estimation

Again, this algorithm is similar to the first. The input consists of only one model 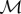 and data 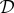. It is assumed that there are unknown parameters and the goal is to find values of these parameters producing the best fit between 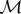 and 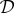. The outputs of steps 2 and 3 need to be adjusted appropriately. The output of step 4 is simply (*â*, *x̂*, *ẑ*), because there is no *α* to be used for rejection. The method also simultaneously estimates hidden/unknown variables and ‘de-noised’ outputs.

**Figure 1.**
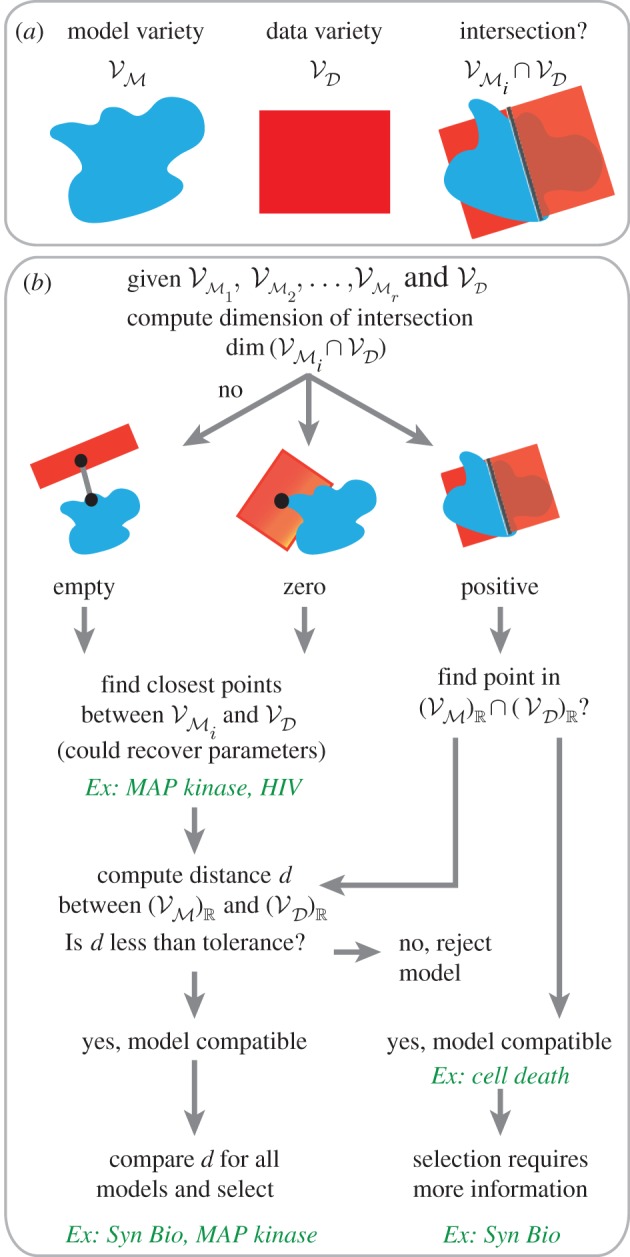
Schematic of numerical algebraic geometry framework corresponding to algorithms 1–3. (*a*) Input to algorithms include model (system of polynomials) translated into a model variety (red), and steady-state data translated into a data variety (blue). (*b*) Flow chart of model compatibility, parameter estimation and model selection methods. Examples (green) are described in §4.

#### 3.2.4. Simple example

**Figure 2.**
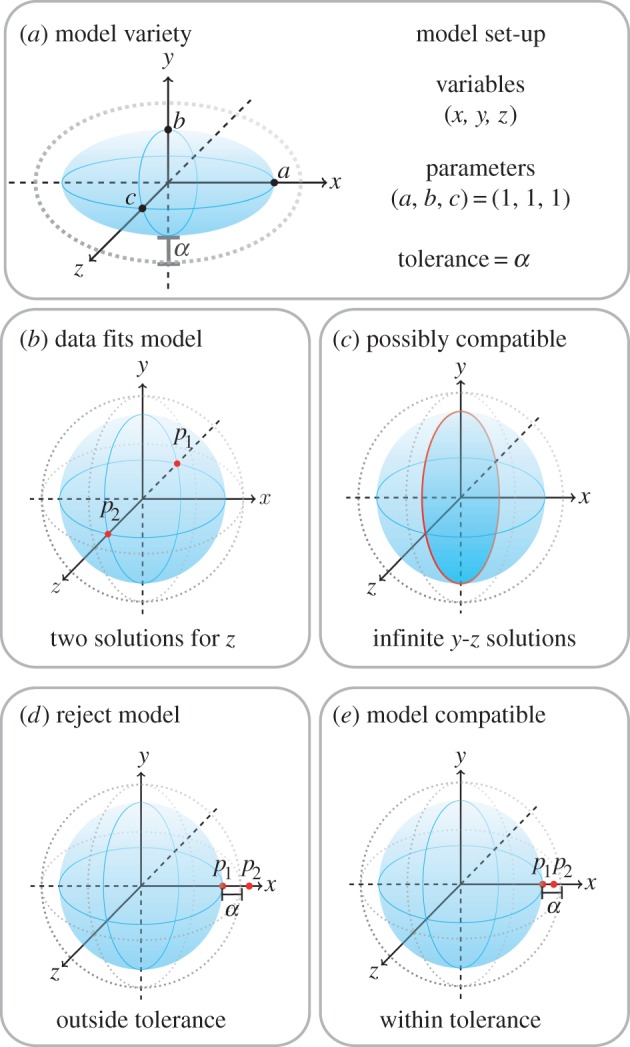
Simple example demonstrating model compatibility following algorithm 1. For ease of illustrating the main idea, we use α instead of 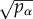 in this figure.

To illustrate algorithm 1, consider a simple model with three variables *x*, *y*, *z* and three parameters *a*, *b*, *c* satisfying
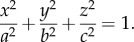
;Let *α* = 0.1 and assume, for this example, that the variances on the errors are 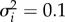 for *i* = 1, 2, 3. The model variety 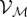 is simply an ellipsoid (figure 2*a*) where *a*, *b*, *c* describe the principal axes. Suppose we know that *a*, *b*, *c* = 1. For the case when the outputs are *x* and *y* and their observed values are *x*′ = 0, *y*′ = 0 and *α* = 0.1, step 2 indicates that the data *do* fit the model, i.e. the model is compatible with the data. In this case, there are two real points in the zero-dimensional intersection 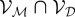 (figure 2*b*). For the same set-up, but with data *x*′ = 0, step 3 indicates that these data *possibly* fit this model. Because there is a positive-dimensional intersection (figure 2*c*), it is possibly compatible (depending on constraints imposed by the user). For the same model and *α*, but different data *x*′ = 1.7, *y*′ = 0, step 3 yields points (1, 0, 0) and (1.7, 0, 0), so the observed value of *d*^2^ is greater than *p_α_* = 0.4605, and the model is rejected (figure 2*d*). However, when the data are *x*′ = 1.01, *y*′ = 0, algorithm 1 indicates model compatibility (figure 2*e*). Previous algebraic methods that required Gröbner basis calculations would result in an empty ideal and thus those approaches are not useful here.

## 4. Results

We demonstrate our methods on problems in biomedicine: cell death activation, synthetic biology, epidemiology and multisite phosphorylation. Each of the forthcoming applications can be written as a mass-action chemical reaction network, which has the form 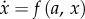, studied at steady state: *f*(*a*, *x*) = 0 as in the problem statement. Throughout these real-world examples, we emphasize the pivotal computations in these methods, such as determining the dimension of the intersection 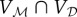 and finding points in the intersection (figure 1*b*). Our first two examples, cell death signalling and genetic toggle biocircuits, demonstrate how to handle positive-dimensional intersections using two different approaches, whereas the remaining two examples, HIV and MAP kinase signalling, highlight analysis of zero-dimensional and empty intersections. In the following examples, we are interested in results that can be interpreted biologically; therefore, we restrict our analysis to non-negative real solutions. We also compare our method results to the squeeze-and-breathe parameter estimation optimization algorithm [35].

### 4.1. Cell death activation

We demonstrate model compatibility (algorithm 1) on an example from receptor-mediated programmed cell death, which is initiated by the activation of death receptors upon the detection of extracellular death ligands [36–39]. We consider, in particular, the ‘cluster’ model of [12], which was inspired by crystallographic data [40] and describes the recruitment of receptors by ligands into local self-activating clusters capable of bistability.

The cluster model is a system of three degree-four polynomials in the form of (2.2) in three variables (representing various receptor states) and six rate parameters, supplemented by ligand and receptor conservations (see the electronic supplementary material). We assume that we can measure the total ligand and receptor concentrations, which may be considered experimental inputs, as well as the concentration of active receptors. We do not assume access to the concentrations of other individual receptor states nor to any of the rate parameters.

A steady-state data point was simulated from the model with all parameters and initial concentrations drawn independently and identically from the lognormal distribution 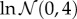, then combined and corrupted with i.i.d. noise from 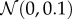 to obtain *y*. The real model and real data varieties intersect in the positive orthant with a distance zero and hence the model is indeed compatible with the data. As detailed in §4.1 of the electronic supplementary material, these runs required a total of approximately 15 s (averaged over 20 runs). The ED degree defined in §3 is 5 for this problem.

### 4.2. Synthetic biology and experimental design

We demonstrate an example from synthetic biology with excess intersection (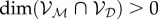). A goal in synthetic biology is to design or modify existing biological systems with new features according to specific design criteria. Reverse engineering of biological systems often includes modules (such as feedback loops), and how these are interconnected are described by different circuits (models). Understanding differences between biocircuit implementations is crucial; therefore, we compare three bistable biocircuits models analysed in [41]: monomer–dimer toggle circuit (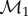), dimer–dimer toggle circuit (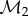) and single-operator gene circuit (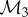), which were initially presented in [42–44]. The model variables include genes (*X_i_*) and proteins (*P_i_*), where *i* = 1 for 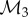 or *i* = 1, 2 for 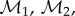 and their species complexes (e.g. *P_i_P_i_*, *X_j_P_i_P_i_*). These variables interact following mass-action kinetics and form systems of polynomial differential equations where 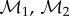 and 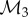 have 7, 8 and 6 model variables, respectively, and 10, 12 and 9 kinetic parameters, respectively. The models can be reduced (given in the electronic supplementary material) by assuming that the total amount of gene 1 (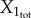) and gene 2 (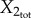) is conserved.

Suppose that the total amounts 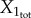 and 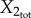 and specific protein synthesis and degradation parameters 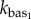, 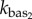, 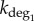 and 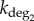 are known. Because protein concentrations are often measurable, we assume that our data are *P*_1_, *P*_2_, and their complexes *P*_1_*P*_1_ and *P*_2_*P*_2_. The aim is to select the best model 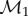, 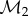 and 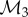 given the data. We simulate steady-state data from the dimer–dimer toggle model (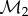) and add Gaussian noise from 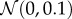. We find that all three have positive-dimensional intersections, where the dimension of the intersections are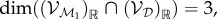;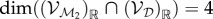;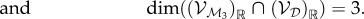;Clearly, all three models are compatible with the data, thus one can only select a ‘best fit’ model using data-independent measures, e.g. number of parameters, dimension, etc.

In fact, the dimensions of the model and data varieties can help us design more informative experiments for model selection. This dimension calculation provides guidance towards the minimal number of additional variable and parameter values that must be measured to ensure 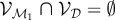. For example, because 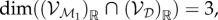 at least four more variables and parameter values must be known. Because we get positive-dimensional intersections, the model is non-identifiable for steady-state data.

Suppose we can experimentally measure four forward biochemical reaction rate constants (e.g. *k_cF_*, *k_kF_*, *k_nF_* and *k_kR_*), then 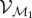 is cut down by four dimensions and does not intersect 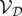. We get similar results for the model varieties associated with 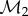 and 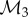 provided we measure rate constants specific to these models (see electronic supplementary material). Now that all the intersections are empty, we run algorithm 2 and find that the sums of squares (equation (3.7)) for each model are as follows: 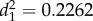, 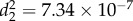 and 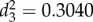. Therefore, we select the 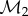 model, which is indeed the true model. As described in the electronic supplementary material, solving the zero-dimensional system for the monomer–dimer toggle circuit, 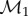, took 1 min and 3 s, solving the system for the dimer–dimer toggle circuit, 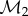, took 1 min and 43 s, and solving the system for the single-operator positive feedback circuit, 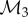 took 0.092 s. The ED degrees as described in §3 are, respectively, 3, 4 and 4.

We compare our results using the squeeze-and-breathe evolutionary optimization algorithm [35]. This method uses Monte Carlo simulation based on an initial parameter ‘prior’ to find local minima of the sum of squared errors (*d*^2^) using derivative-free optimization. At the end of each iteration, it computes a ‘posterior’ from the best local minima, which is then used as a ‘prior’ in the next iteration. The sample–optimize–recompute cycle continues until convergence. One advantage is that the local optimization steps allow it to explore beyond the ‘prior’ (i.e. the true parameter value could lie outside of it) [35], which is ideal, because the NAG method does not restrict to certain ranges of the parameter space. Moreover, squeeze-and-breathe has good success finding the true parameter (global minimum) in biological models [45] and therefore is a suitable choice to compare with our NAG method. As before, we take data from 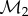 and estimate parameters based on the previous experimental design analysis. We estimate parameters 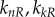 for 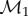, 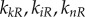 for 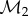 and 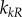 for 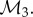 We then get the sum of squared errors for the best-fit parameter of each model: 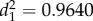, 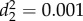, 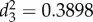. The method takes approximately 10 min to run for each model and also correctly selects 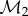.

### 4.3. Human immunodeficiency virus progression

We demonstrate parameter estimation (algorithm 3) on an example coming from epidemiology. We use a model that includes long-term HIV dynamics from initial viremia, latency and virus increase [46], based on [47]. In the model (see the electronic supplementary material), the HIV virus inhibits the CD4^+^ T cell population while promoting macrophage proliferation, which, in turn, houses the replicating virus. As macrophages proliferate, the virus reservoir increases, so the model offers a description of HIV patient progression to acquired immunodeficiency syndrome. Model variables *x* are uninfected CD4^+^ T cells (*T*), infected CD4^+^ T cells (*T_i_*), uninfected macrophages (*M*), infected macrophages (*M_i_*) and HIV virus population (*V*).

Hernandez-Vargas *et al*. show that the model can have two real equilibria, one of which is stable, representing patients that are ‘long-term non-progressors’ [46]. The parameters *a* are 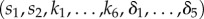, where *s_i_* represents synthesis of T cells and macrophages, *k*_*i*_ are rate constants describing interactions between variables *x*, and *δ_i_* represents natural death. For this example, *y* = *x*. We estimated the natural death of the virus, parameter *δ*_5_, using the long-term non-progressors steady-state value (table 3 of [46]) and adding noise to each variable 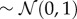. By algorithm 3, the data variety and model variety do not intersect. We find the closest point and estimate 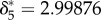 (true value of *δ*_5_ = 3, which was obtained by conferring with the authors of [46]). This run took 48 s, as described in the electronic supplementary material. The relevant ED degree is 16.

### 4.4. Multisite phosphorylation with experimental data

We examine phosphorylation mechanisms of cellular signalling with experimental data, and demonstrate model selection (algorithm 2) and parameter estimation (algorithm 3). We focus on phosphorylation, a key cellular regulatory mechanism that has been the subject of extensive study, both experimentally and theoretically ([48] and references therein). An area of interest is the mechanism by which a kinase phosphorylates a two-site substrate, either distributively, where the kinase can add at most one phosphate before dissociating, or processively, where it can add both phosphates in sequence. The MAPK/ERK pathway is a well-known system for studying phosphorylation, whereby MEK (kinase) phosphorylates ERK (its substrate). Aoki *et al*. [49] showed experimental evidence, while working with polynomial models, that the mammalian MAPK/ERK pathway acts distributively *in vitro* but processively *in vivo*.

We compare these distributive and processive models against the *in vivo* data reported in the same study. The distributive model consists of 12 molecular species and 17 mass-action reactions, whereas the processive model has 14 species and 18 reactions (species correspond to variables, each reaction corresponds to a parameter). The data take the form of 36 concentration measurements of three aggregate phosphoforms over a range of 12 EGF stimulation levels. All model parameters are calibrated using *in vitro* estimates by [49], except the parameter *k*_1_ representing EGF loading, which we estimate for both models (see electronic supplementary material).

**Figure 3.**
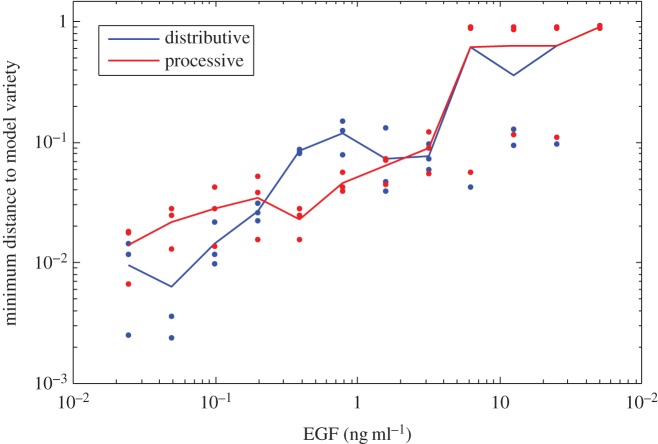
MAP kinase model selection using 36 data points from three model variables (aggregate phosphoforms of ERK) taken in triplicate at each of 12 EGF stimulation levels.

Next, we perform model selection by running algorithm 2 on each data point individually and select independently for each run the preferred model (more details in the electronic supplementary material). Under low EGF stimulation, the best model estimates are nearly identical with a slight preference for distributive. At high EGF stimulation, the models are identical with no preference for one model over the other. These results can be justified by noting that the main distinction between distributivity and processivity is nonlinear switching behaviour (i.e. a sigmoidal response curve), and this occurs only at intermediate stimulations. However, at medium EGF stimulation (figure 3), there is a preference for the processive model, which supports the findings in [49]. As detailed in §4.4.3 of the electronic supplementary material, all runs for the distributive model took approximately 77 s, whereas the larger processive model required about 131 s (both averaged over 20 runs). It is worth noting that each of the 36 parameter homotopy runs in each of the two cases took less than 2% of the time of a regular, non-parameter homotopy run (less than 1% for the processive model). The ED degree for this problem is 20 for both models.

## 5. Conclusion

The problem of determining whether given real-world data fit one or more given mathematical models is challenging. When a model is defined by algebraic (polynomial) functions, the methods of NAG may be employed to study the geometry underlying the model and data. In particular, these methods are useful for model variables observed at steady state. We demonstrated this numerical and geometric framework for comparing models with experimental dose–response data in MAPK/ERK pathway and highlighted that the intermediate EGF doses were the most informative for model selection, complementing another finding that model selection results can be very sensitive to experimental parameters [50].

Despite the difficulties associated with positive-dimensional components and limitations in analysis, we can reproduce compatible models, and furthermore, can predict additional information, such as measurements of parameters, that are necessary for selecting models. Our geometric investigations of positive-dimensional components may perhaps relate to algebraic analyses for biochemical models, such as model identifiability or matroids for experimental design [13,51,52].

There are further directions to be considered in this vein, aside from making the existing computational methods more efficient. First, there would be great value in developing strictly *real* geometric methods for solving polynomial systems such as those that appear in this article. Some such techniques exist, but only in very special circumstances. Second, there would be much value in developing effective numerical methods for treating inequalities. It should be noted that the methods described in [53] and the references therein will incorporate such constraints, though the cost of such computations restricts their use to relatively low dimensions. Finally, there is likely much to be gained from considering the geometry underlying models not defined by algebraic functions. Algebraic geometry provides very clean, well-understood structures, paving the way for numerical methods. Differential geometry or topology could lead to similarly useful techniques for model selection and parameter estimation.

## 6. Material and methods

### 6.1. Numerical algebraic geometry

General references for NAG include [17,29], with the latter doubling as a user manual for the software package Bertini. For computations, we used Bertini 1.4 and Macaulay2 v. 1.6.

### 6.2. Data generation

Data simulated from cell death activation, synthetic biology and HIV models were performed in Matlab R2014b using ode15s.

## Supplementary Material

Supplementary Material for: Numerical algebraic geometry for model selection and its application to the life sciences
