# Supplementary material for: Numerical algebraic geometry for model selection and its application to the life sciences
Source: J R Soc Interface. 2016 Oct;13(123):20160256. doi: 10.1098/rsif.2016.0256 (PMC5095207; doi:10.1098/rsif.2016.0256)
Supplement: Supplementary Material for: Numerical algebraic geometry for model selection and its application to the life sciences [file rsif20160256supp1.pdf]

Supplementary Material for: Numerical algebraic geometry for model selection and its application to the life sciences

Elizabeth Gross[1], Brent R. Davis[2], Kenneth L. Ho[3], Daniel J. Bates[2], Heather A. Harrington[4]
[1]Department of Mathematics, San José State University,
[2]Department of Mathematics, Colorado State University,
[3]Department of Mathematics, Stanford University,
[4]Mathematical Institute, University of Oxford.

Contents

1 Geometry **1**
1.1 Numerical algebraic geometry for isolated solutions . . . 1
1.2 NAG for positive-dimensional solution sets . . . 2
1.3 Software for NAG . . . 3
1.4 Geometry specific to presented algorithms . . . 3

2 Choice of test statistics and parameter estimates **3**
2.1 Maximum likelihood . . . 3

3 Algorithm modifications **5**
3.1 Solving the constrained optimization problem . . . 5
3.2 Removing extinction components . . . 6

4 Results **6**
4.1 Cell death activation . . . 7
4.2 Synthetic biology and experimental design . . . 11
4.3 Epidemiology HIV . . . 13
4.4 Multisite phosphorylation . . . 15
4.4.1 Biology of MAP Kinase system . . . 15
4.4.2 Mathematical models . . . 16
4.4.3 Model selection and parameter estimation computations . . . 18

1 Geometry

In this section we briefly review the some of the fundamental geometric concepts needed for the methods in the main text.

1.1 Numerical algebraic geometry for isolated solutions

Numerical algebraic geometry (NAG) refers to the use of numerical methods, particularly homotopy continuation-based methods, to compute approximations to solutions of polynomial systems. In other words, given a

polynomial system $F : \mathbb{C}^N \to \mathbb{C}^n$ with n equations in N variables, NAG seeks to find numerical approximations of all $z \in \mathbb{C}^N$ such that $F(z) = 0$. It may be the case that the solution set of F has infinitely many such points (curves, surfaces, etc.), in which case the data structure that encodes the solutions is called a *witness set*. See the description of the *numerical irreducible decomposition* in Section 1.2 for more on this. For simplicity, we assume in this section that $N = n$. The books [1, 2] and the references therein provide additional explanations from a mathematical and computational perspective.

The core technique for most algorithms is *homotopy continuation*. The idea of homotopy continuation is to cast the polynomial system to be solved, say $F : \mathbb{C}^N \to \mathbb{C}^N$, as a member of a parameterized family of polynomial systems, $H : \mathbb{C}^N \times \mathbb{C} \to \mathbb{C}^N$, called a homotopy $H(z,t)$ with parameter $t \in \mathbb{C}$, that includes one polynomial system $G : \mathbb{C}^N \to \mathbb{C}^N$ that is easily solved and has the special property of having "enough" isolated solutions. In this document, we use the `Bertini` [3] convention that $H(z,1) = G(z)$ and $H(z,0) = F(z)$, i.e., t marches from 1 to 0. There are several canonical options for the construction of such a homotopy, and the reader is encouraged to consult [1, 2] and the references therein for further details.

As t varies from 1 to 0, some results from algebraic geometry tell us that the solutions of the polynomial system $H(z,t) = 0$ vary continuously and generally stay distinct until $t = 0$, where they may converge to solutions of $F(z) = 0$ or diverge. More specifically, there is a measure zero subset of $t \in \mathbb{C}$, meaning a finite set of points in this particular parameter space, over which two or more solutions coalesce. Such occurrences are thus probability zero events and, furthermore, can be detected on the fly and avoided. Said more technically, there is a *Zariski open, dense* set of the parameter space above which the solution set is finite and consists of a fixed number of solutions. Here, "Zariski" refers to the *Zariski topology*, for which basic open sets are the complements of solution sets of polynomial systems.

In practice, t is moved in discrete increments, not continuously. For each solution at $t = 1$, a path of solutions is *tracked* using numerical predictor/corrector methods as t advances to 0. Implementations typically utilize adaptive step lengths and adapative precision. There are far too many details about this procedure to give a thorough explanation here. Instead, refer to the books for further details.

Ultimately, the output of this procedure is a superset of numerical approximations of the isolated solutions of $F(z) = 0$, possibly including approximations to points lying on positive-dimensional components, if any. It is important to note that this procedure necessarily works over $\mathbb{C}$ and finds all complex solutions. Real solutions could be buried somewhere within the complex solutions, and it is particularly difficult to extract these outside of the zero-dimensional case. However, methods do exist to extract such a real point [4–6]; here we use the method of [6], which is guaranteed (with probability one) to minimize the distance of a prescribed real point to each real connected component of the solution set defined by $F(z) = 0$.

1.2 NAG for positive-dimensional solution sets

For solution sets of positive dimension (curves, surfaces, etc.), there is an extension of homotopy continuation referred to as the *numerical irreducible decomposition (NID)*. As opposed to the case of systems of linear equations (at most one solution component of one dimension), there may be many components of many different dimensions. For example, one solution set might consist of seven components of dimension four, five surfaces, three curves and 15 isolated points. Furthermore, components may be singular, meaning that the Jacobian matrix is rank-deficient throughout the component.

Technical definitions used in NAG such as *degree*, *dimension*, and *irreducible component* go a bit beyond the scope of this paper. It is enough to know that each "piece" of a solution set has a fixed complex dimension (e.g., a curve has dimension one, a surface two, etc.) and by the *dimension* of a solution set of a polynomial system of equations, we mean the maximum of the dimensions of the irreducible components.

The *NID* of a solution set of a polynomial system consists of a catalog of the dimensions and degrees of each irreducible component, along with a set of *witness points* on each component. By *degree*, we mean the number of points in the intersection of a component with a randomly-chosen affine linear space of complementary dimension. The *witness points* on a component are then exactly these points (and thus depend on the choice of linear space). One fundamental result from algebraic geometry is that an irreducible component will *almost* always intersect a complementary-dimensional linear space exactly in a set of points *and* that the number of points is the same for *almost* any choice of linear space. Again, this can be stated as a probability one guarantee or with Zariski open, dense sets, but we choose not to be that technical here.

1.3 Software for NAG

Various NAG software packages have been produced over the years. Currently, there are three main options: `PHCpack` [7], `HOM4PS-3` [8], and `Bertini` [3], each with their own benefits and drawbacks. In this article, we used `Bertini` exclusively.

1.4 Geometry specific to presented algorithms

Proposition 1 of the main text should be familiar to those trained in multivariate calculus; this is essentially the method of Lagrange multipliers. Geometrically, this system of equations forces the gradient of the objective function to be perpendicular to the tangent space of the model variety.

When working with an irreducible component of the solution set of a system of polynomial equations, it is often useful to deal with a *complete intersection*. Said simply, the idea is that computations can be more difficult if there are more equations than necessary. To see why this might be true, let us consider an example from linear algebra. Suppose we have a single linear equation in three variables, defining a plane. Now suppose we consider a system of two equations consisting of that equation and twice that equation (having the same solution set). Then the matrix of coefficients of this linear system is not full rank (not a desirable situation) and we have two equations defining a geometric object that could be described by a single equation.

In the nonlinear setting, the situation is quite similar. Having "too many" equations leads to an undesirable rank-deficient Jacobian matrix. Suppose polynomial system $F : \mathbb{C}^N \to \mathbb{C}^n$ has an irreducible component X of dimension $N - m$ (*codimension* m). Then, again with probability one, the polynomial system $A \cdot F$ has X as an irreducible component but has the "correct" number of equations, where A is a random constant matrix with n columns and m rows. Here, "correct" means the number of equations matches to codimension m. We will refer to this method as *squaring up*.

Finally for this section specific to the algorithms developed in the main text, we require the user to check two geometric facts, the meaning of which may not be entirely clear.

1. $\mathcal{V}_\mathcal{M} \cap \mathcal{V}_\mathcal{D}$ refers to the intersection of the model and data varieties, as defined in the main text. To find the intersection of two solution sets, it is sufficient to simply solve the system consisting of all equations appearing in the systems for $\mathcal{V}_\mathcal{M}$ and $\mathcal{V}_\mathcal{D}$, i.e., the union of those two polynomial systems. There are more sophisticated methods, but this is sufficient.

2. The user must determine whether the intersection of $(\mathcal{V}_M)_\mathbb{R}$ and $(\mathcal{V}_M)_\mathbb{R}$ is empty. By this, we simply mean that one should search for real points in the intersection just described, e.g., using the method of [6].

2 Choice of test statistics and parameter estimates

In this section, we justify our procedures for model validation and parameter estimation.

2.1 Maximum likelihood

Here we justify the assertion that the test statistic given in Section 2.1 of the main text is related to likelihood maximization.

Consider first, for simplicity, the case of a single data point $y = (y_1, \ldots, y_m)$, which we assume is a perturbation $y = \xi + \epsilon$ of some unknown true value $\xi = (\xi_1, \ldots, \xi_m)$, where each component ϵ_i of the error $\epsilon = (\epsilon_1, \ldots, \epsilon_m)$ is an independent zero-mean Gaussian random variable with variance σ_i^2. We are interested in computing the probability that y comes from a given model as defined by a model variety $\mathcal{V}_\mathcal{M}$. A point on $\mathcal{V}_\mathcal{M}$ has the form (a, x, z), where $a = (a_1, \ldots, a_k)$ are the model parameter values, $x = (x_1, \ldots, x_n)$ are the variable values, and $z = (z_1, \ldots, z_n)$ are the outputs. The probability that y comes from a given point

$(a, x, z) \in \mathcal{V}_{\mathcal{M}}$, i.e., that y is a perturbation of z where $(a, x, z) \in \mathcal{V}_{\mathcal{M}}$ for some a and x, is then:

$$\Pr(y \mid a, x, z) = \Pr(y \mid \xi = z) = \prod_{i=1}^{m} \Pr(y_i \mid \xi_i = z_i).$$

This is also called the likelihood $L(a, x, z \,|\, y)$ of (a, x, z) and we wish to find its maximizer over all $(a, x, z) \in \mathcal{V}_{\mathcal{M}}$. This can equivalently be done by considering the log-likelihood, which gives:

$$\log L(a, x, z \mid y) = \sum_{i=1}^{m} \log \Pr(y_i \mid \xi_i = z_i) = \sum_{i=1}^{m} \left(\frac{1}{2} \log 2\pi\sigma_i^2 - \frac{(z_i - y_i)^2}{2\sigma_i^2} \right)$$

by normality. The maximizer $(\hat{a}, \hat{x}, \hat{z})$ can therefore be found by solving the optimization problem:

$$d^2 = \min_{(a,x,y) \in \mathcal{V}_{\mathcal{M}}} \sum_{i=1}^{m} \frac{(z_i - y_i)^2}{\sigma_i^2},$$

where the optimum is precisely the test statistic. The values $\hat{a}$, $\hat{x}$, and $\hat{z}$ are the maximum likelihood estimates for, respectively, the parameters (estimation), the unobservable variable values (inference/recovery), and the true output values (filtering/denoising),

The test statistic d^2 itself also has a useful interpretation as follows. Suppose that y comes from a point $(a, x, z) \in \mathcal{V}_{\mathcal{M}}$. Then:

$$d^2 = \sum_{i=1}^{m} \frac{(\hat{z}_i - y_i)^2}{\sigma_i^2} \leq \sum_{i=1}^{m} \frac{(z_i - y_i)^2}{\sigma_i^2}$$

by definition. But regarding each y_i as a random variable, each term $(z_i - y_i)/\sigma_i$ in the summation above is standard normal. Hence the right-hand side has a chi-squared distribution with m degrees of freedom (χ_m^2). The inequality should be interpreted by regarding d^2 as a random variable subject to the same source of randomness. This can be written somewhat clearer as:

$$d^2(\omega) \leq \sum_{i=1}^{m} \frac{(z_i - y_i(\omega))^2}{\sigma_i^2},$$

where we have made explicit the underlying dependence of both sides on the same random realization ω. The inequality then holds for each value of ω. Consequently, we conclude that:

$$\Pr(d^2 \leq u) \geq \Pr(U \leq u), \quad U \sim \chi_m^2,$$

so

$$\Pr(d^2 \geq p_\alpha) \leq \Pr(U \geq p_\alpha) = \alpha, \quad U \sim \chi_m^2,$$

where p_α is the upper α-percentile for χ_m^2. This can be used to test the hypothesis that y comes from $\mathcal{V}_{\mathcal{M}}$. The test statistic is also related to the log–maximum-likelihood as:

$$\log L(\hat{a}, \hat{x}, \hat{z}) = \frac{1}{2} \left(m \log 2\pi + \sum_{i=1}^{m} \log \sigma_i^2 - d^2 \right),$$

which is a useful quantity for model selection via, e.g., the Akaike or Bayesian information criteria.

Now consider the case of multiple data points $\{y^{(j)}\}_{j=1}^{p}$. As before, we assume that each $y^{(j)} = (y_1^{(j)}, \ldots, y_m^{(j)})$ is a perturbation $y^{(j)} = \xi^{(j)} + \epsilon^{(j)}$, where each $\epsilon_i^{(j)}$ is an independent zero-mean Gaussian random variable with variance $\sigma_{j,i}^2$. Instead of searching for one point on $\mathcal{V}_{\mathcal{M}}$, we now have to search for p points $(a, x^{(j)}, z^{(j)})$ for $j = 1, \ldots, p$ all with the same parameter values (since they come from the same fixed model realization). The probability that $y^{(j)}$ comes from $(a, x^{(j)}, z^{(j)})$ for $j = 1, \ldots, p$ is then:

$$\begin{aligned} \Pr(y^{(1)}, \ldots, y^{(p)} \mid a, x^{(1)}, z^{(1)}, \ldots, x^{(p)}, z^{(p)}) = \prod_{j=1}^{p} \Pr(y^{(j)} \mid \xi^{(j)} = z^{(j)}) &= \prod_{j=1}^{p} \prod_{i=1}^{m} \Pr(\hat{x}_{j,i} \mid \xi_i^{(j)} = z_i^{(j)}) \\ &\equiv L(a, x^{(1)}, z^{(1)}, \ldots, x^{(p)}, z^{(p)}) \end{aligned}$$

by independence. This is essentially the same as before except that we now loop over each coordinate of each data point. Therefore, the maximum likelihood estimates $(\hat{a}, \hat{x}^{(1)}, \hat{z}^{(1)}, \dots, \hat{x}^{(p)}, \hat{z}^{(p)})$ can be obtained by solving:

$$d^2 = \min_{(a,x^{(1)},z^{(1)},\dots,x^{(p)},z^{(p)})\in\mathcal{V}_\mathcal{M}} \sum_{j=1}^{p}\sum_{i=1}^{m} \frac{(z_i^{(j)} - y_i^{(j)})^2}{\sigma_{j,i}^2}.$$

The same arguments go through and we find that $\Pr(d^2 \geq p_\alpha) \leq \alpha$ for p_α the upper α-percentile for χ^2_{mp}. The log–maximum-likelihood is related to d^2 as:

$$\log L(\hat{a}, \hat{x}^{(1)}, \hat{z}^{(1)}, \dots, \hat{x}^{(p)}, \hat{z}^{(p)}) = \frac{1}{2}\left(pm\log 2\pi + \sum_{j=1}^{p}\sum_{i=1}^{m}\log\sigma_{j,i}^2 - d^2\right).$$

3 Algorithm modifications

The algorithms presented in the main text are in their simplest form. Some applications require modifications, particularly if there are constraints on the variables or parameters.

3.1 Solving the constrained optimization problem

In many settings, there exist constraints on the variable and parameter spaces. For example, in chemical reaction networks, the rate parameters are all assumed to be nonnegative. Thus, when nonnegativity or other constraints are present, instead of finding the weighted squared distance between two varieties, we are finding the weighted squared distance between two *semi-algebraic sets*, i.e. sets defined by polynomial equalities and inequalities as opposed to just polynomial equalities. Indeed, if we let $S_\mathcal{M} \subset (\mathcal{V}_\mathcal{M})_\mathbb{R}$ denote the semi-algebraic set associated to the model, e.g. $S_\mathcal{M} = \mathcal{V}_\mathcal{M} \cap \mathbb{R}^{k+n+m}_{\geq 0}$, then the appropriate statistic is:

$$d^2 = \min \sum_{i=1}^{m} \frac{(z_i - y_i)^2}{\sigma_i^2} \text{ subject to } (a, x, z) \in S_\mathcal{M}.$$

In the case when a bound on the statistic d^2 is sufficient, then no additional work is needed. One can solve the system from Proposition 1, keeping in mind that the weighted squared distance between the closest pairs of points returned would be an upper bound on d^2. If the closest point to $(\mathcal{V}_\mathcal{D})_\mathbb{R}$ in $(\mathcal{V}_\mathcal{M})_\mathbb{R}$ is also an element of $S_\mathcal{M}$, then the squared distance would be exactly the statistic d^2.

When the exact value of d^2 is needed, then one should solve the Fritz John (FJ) system of equations. Let $F_1, \dots, F_r$, $h_1, \dots, h_s$ be polynomials in the ring $\mathbb{R}[a_1, \dots, a_k, x_1, \dots, x_n, z_1, \dots, z_m]$. Let $S_\mathcal{M}$ be the semi-algebraic set of all $(a, x, z) \in \mathbb{R}^{k+n+m}$ that satisfies:

$$F_i(a, x, z) = 0 \text{ for } i = 1, \dots, r$$
$$h_i(a, x, z) \leq 0 \text{ for } i = 1, \dots, s.$$

Let $\lambda_0, \lambda_1, \dots, \lambda_r, \mu_1, \dots, \mu_s$ be indeterminates (these are the FJ multipliers). The FJ system may be written as:

$$F = 0 \tag{1}$$
$$\lambda_0 \begin{bmatrix} 0 \\ z - y \end{bmatrix} + \sum_{i=1}^{r} \lambda_i \nabla_{a,x,z} F_i + \sum_{i=1}^{s} \mu_i \nabla_{a,x,z} h_i = 0 \tag{2}$$
$$\mu_1 h_1 = 0 \tag{3}$$
$$\vdots \tag{4}$$
$$\mu_s h_s = 0. \tag{5}$$

If (a^*, x^*, z^*) is a critical point the FJ constraint qualification states that there exists a *nonzero* vector $[\lambda_0, \ldots, \lambda_r, \mu_1, \ldots, \mu_s]$ with $\mu_i \geq 0$ for $i = 1, \ldots, s$ so that $((a^*, x^*, z^*), [\lambda_0, \ldots, \lambda_r, \mu_1, \ldots, \mu_s])$ satisfies equations (1)–(5). Thus, we can find the global minimum by using NAG to solve the system defined by equations (1)–(5), then filtering solutions appropriately. This combination of NAG and optimization equations (KKT in that case) was first employed in [9].

In some situations, it may be more efficient (or necessary) to minimize the objective function over $(\mathcal{V}_\mathcal{M})_\mathbb{R}$ and then check the boundaries of $\mathcal{S}_\mathcal{M}$. This removes the explicit inequality constraints $h_i(a, x, z) \leq 0$. This approach is needed in the case when F is not a complex intersection. Our approach is to use a complex matrix A to construct a polynomial system $A \cdot F$ whose solutions contain solutions of F. In this case, it no longer makes sense to construct the system (1)–(5).

We describe this method for when there are only nonnegative constraints on each indeterminate. Following the notation of Proposition 1 in the main text, assume that $h(a, x, y)$ are equations that define a complete intersection whose solutions contain $\mathcal{V}_\mathcal{M}$. First, solve the equality constrained optimization problem on $(\mathcal{V}_\mathcal{M})_\mathbb{R}$ using the FJ system in Proposition 1. This will provide an upper bound for d^2 defined on $\mathcal{S}_\mathcal{M}$. The space $\mathbb{R}^{k+n+m}_{\geq 0}$ is naturally a convex polytope made up of faces of various dimensions. Each of the j faces in dimension i, $\mathcal{F}_{i,j}$, is contained in its affine hull $\overline{\mathcal{F}_{i,j}}$, or smallest affine space that contains that face. In our special case, $\overline{\mathcal{F}_{i,j}}$ is defined simply by imposing natural equality constraints. We then minimize d^2 over $\mathcal{V}_\mathcal{M} \cap \overline{\mathcal{F}_{i,j}}$ for each combination of i, j and then filter out solutions not contained in $\mathcal{V}_\mathcal{M} \cap \mathbb{R}^{k+n+m}_{\geq 0}$. This is equivilent to minimizing d^2 over $S_\mathcal{M}$.

In total, if there are N indeterminates, there are $2^N - 1$ faces. This amount to solving $2^N - 1$ FJ systems. One observation is that the number of systems that need to be solved explodes when N is large. However, the dimension of $\mathcal{V}_\mathcal{M} \cap \overline{\mathcal{F}_{i,j}}$ is less than or equal to the dimension of $\mathcal{V}_\mathcal{M}$, with the inequality being strict when $V_\mathcal{M} \subsetneq \overline{\mathcal{F}_{i,j}}$. If $\mathcal{V}_\mathcal{M} \cap \overline{\mathcal{F}_{i,j}}$ is empty, then $\mathcal{V}_\mathcal{M} \cap \mathcal{S}$ is also empty for any subset $S \subset \overline{\mathcal{F}_{i,j}}$. Using this fact, we significantly reduce the number of lower-dimensional affine spaces that need to be checked since they are composed of intersections of higher-dimensional affine spaces.

For example, in Section 4.4.3, the MAP Kinase model variety $\mathcal{V}_\mathcal{M}$ is one-dimensional $\mathcal{V}_\mathcal{M}$ and in each case where we intersect $V_\mathcal{M}$ with affine space defined by a_i, x_i, or z_i set to zero, the intersection consists of a finite set of isolated points. Further analysis shows that 16 faces are sufficient to minimize d^2 over $\mathcal{S}_\mathcal{M}$ even though there are $2^{16} - 1 = 65,535$ faces to check.

3.2 Removing extinction components

Given a model, it is quite common that the model variety is not irreducible but instead is the union of several irreducible components. In applications, it may be preferred to remove from consideration components that lie entirely in a coordinate hyperplane, since, in such components, one or several of the parameters and/or variables are equal to zero. For example, in a chemical reaction network, such a component is called an *extinction component* [10] since it captures the situation where one or more of the reactants have "run out." It is common to want to avoid extinction components when estimating parameters.

Removing components where a parameter or variable is equal to zero throughout the set from consideration can be done algebraically with *saturation*. In particular, if $I_\mathcal{M}$ is the defining ideal of the model $\mathcal{V}_\mathcal{M}$ one should compute

$$I_{Main} = I_\mathcal{M} \ : \ (a_1 \cdots a_k \cdot x_1 \cdots x_n \cdot z_1 \cdots z_m)^\infty :=$$

$$\{f \in \mathbb{R}[a_1, \ldots, a_k, x_1, \ldots, x_n, z_1, \ldots, z_m] \ : \ \exists k \in \mathbb{N} \text{ s.t. } (a_1 \cdots a_k \cdot x_1 \cdots x_n \cdot z_1 \cdots z_m)^k f \in I_\mathcal{M}\}.$$

This procedure can be performed using the `saturate` command in `Macaulay2`.

To estimate parameters such that the best estimate corresponds to a point not on an extinction component of $\mathcal{V}_\mathcal{M}$, one should modify Algorithm 3, replacing $\mathcal{V}_\mathcal{M}$ with $\mathcal{V}(I_{Main})$.

4 Results

We provide details for the calculations of examples in the main text. All code is available at:
`http://www.math.sjsu.edu/∼egross/NAGModelSelection/AuxillaryFiles`.

4.1 Cell death activation

We provide the details of the calculations for model compatibility of the cell death cluster model. This subsection includes detailed information regarding the solving schemes available in NAG software that were utilized. A summary for the practioner can be found at the end of the subsection.

The model describes activation of apoptosis by death receptor Fas mechanisms [11]. The model includes constitutive receptor opening and closing, pairwise open Fas stabilization, higher-order open Fas stabilization enabled by FasL, and ligand-induced receptor opening. According to its conformational states, Fas is assumed to be one of three species: closed (X_1); open, unstable (X_2); and open, stable (X_3), i.e., active and signaling. Furthermore, let the ligand FasL be denoted by L. Then the model has the reactions:

$$
\begin{aligned}
X_2 &\underset{k_o}{\overset{k_c}{\rightleftharpoons}} X_1, \\
X_3 &\xrightarrow{k_u} X_2, \\
jX_2 + (i-j)X_3 &\xrightarrow{k_s^{(i)}} (j-k)X_2 + (i-j+k)X_3, \\
L + jX_2 + (i-j)X_3 &\xrightarrow{k_l^{(i)}} (j-k)X_2 + (i-j+k)X_3
\end{aligned}
$$

for $i \in \{2,3\}$, $j = 1,\ldots,i$, and $k = 1,\ldots,j$. The first reaction describes spontaneous receptor opening and closing. The second reaction describes constitutive destabilization of open Fas. The third reaction describes cluster-stabilization by open Fas, independent of the presence of FasL. The fourth reaction describes cluster-stabilization events enabled by FasL.

Assuming mass-action kinetics, the reactions can be translated as follows:

$$
\begin{cases}
\dot{x_1} &= -v_1, \\
\dot{x_2} &= v_1 + v_2 - v_3 - v_4, \\
\dot{x_3} &= v_3 + v_4 - v_2,
\end{cases}
\quad \text{where} \quad
\begin{cases}
v_1 &= k_o x_1 + (-k_c)x_2, \\
v_2 &= k_u x_3, \\
v_3 &= 6k_s^{(3)}x_2^3 + 3k_s^{(3)}x_2^2x_3 + 3k_s^{(2)}x_2^2 + k_s^{(3)}x_2x_3^2 + k_s^{(2)}x_2x_3, \\
v_4 &= 6k_l^{(3)}x_2^3 l + 3k_l^{(3)}x_2^2x_3 l + 3k_l^{(2)}x_2^2 l + k_l^{(3)}x_2x_3^2 l + k_l^{(2)}x_2x_3 l,
\end{cases}
$$

where v_i are the reaction velocities for the variables x_i, and lowercase letters denote the concentrations of their uppercase counterparts.

The model parameters for the cell death cluster model are:

$$a = (k_o, k_c, k_u, k_s^{(2)}, k_s^{(3)}, k_l^{(2)}, k_l^{(3)}),$$

the variables are:

$$x = (\ell, x_1, x_2, x_3),$$

and the outputs are:

$$z = (\lambda, \rho, \zeta)$$

where the outputs represent, respectively, the total ligand concentration, the total receptor concentration, and the total downstream "death signal", as given by the equations:

$$
\begin{aligned}
\lambda - \ell &= 0 && (6) \\
\rho - (x_1 + x_2 + x_3) &= 0 && (7) \\
\zeta - x_3 &= 0. && (8)
\end{aligned}
$$

We set the reaction rates $\dot{x}_1, \dot{x}_2, \dot{x}_3$ to zero, and, together with equations (6)–(8), we obtain defining equations for the model variety $\mathcal{V}_\mathcal{M}$. The ambient space that $\mathcal{V}_\mathcal{M}$ is contained in has dimension 14. This space has coordinates defined by both the model parameters a, the variables x and the outputs z.

Given an observable data point $y = (\lambda', \rho', \zeta')$, we define the data variety as:

$$\mathcal{V}_\mathcal{D} = \{(x, a, z) \in \mathbb{C}^{14} : \lambda = \lambda',\ \rho = \rho',\ \zeta = \zeta'\} \quad (9)$$

for the clustering model. Note that $\mathcal{V}_{\mathcal{D}}$ has dimension 11 since there are no degrees of freedom in the variables λ, ρ, or ζ.

We first compute a numerical irreducible decomposition (NID) of $\mathcal{V}_{\mathcal{M}}$ using `Bertini`; this will aid in understanding $\mathcal{V}_{\mathcal{M}} \cap \mathcal{V}_{\mathcal{D}}$. One can verify, after computing the NID for $\mathcal{V}_{\mathcal{M}}$, that $\mathcal{V}_{\mathcal{M}}$ is a 9-dimensional complex algebraic set of degree 10 (file name: `Cluster_Model_NID`).

Now suppose we are given the following data point (taken from the model without noise):

$$y = (\lambda', \rho', \zeta') = (1.7784308, 2.31883024, 2.16896112).$$

One can then verify using the NID that $\mathcal{V}_{\mathcal{M}} \cap \mathcal{V}_{\mathcal{D}} \neq \emptyset$ (file name: `Cluster_Model_Data_NID`). Specifically, $\mathcal{V}_{\mathcal{M}} \cap \mathcal{V}_{\mathcal{D}}$ is a 6-dimensional complex algebraic set of degree 5. Adding noise to the coordinates of y taken from $\mathcal{N}(0, 0.1)$ did not affect the dimension or degree.

Since we are interested in model compatibility, our goal is to find at least one nonnegative point in $(\mathcal{V}_{\mathcal{M}})_{\mathbb{R}} \cap (\mathcal{V}_{\mathcal{D}})_{\mathbb{R}}$. The above computation at least provides evidence that this is the case, but it may be possible that $\mathcal{V}_{\mathcal{M}} \cap \mathcal{V}_{\mathcal{D}}$ does not contain any nonnegative real points or any real points at all for that matter. We will approach this problem using the methods described in [6]. If $\mathcal{V}_{\mathcal{M}} \cap \mathcal{V}_{\mathcal{D}}$ contains a real nonnegative point then we cannot reject the model and may conclude that the model is compatible with the data. If $(\mathcal{V}_{\mathcal{M}})_{\mathbb{R}} \cap (\mathcal{V}_{\mathcal{D}})_{\mathbb{R}}$ does not contain a real point then we can try and use a Lagrange multiplier method similar to the one employed in Section 4.4.3 in dealing with model selection.

We first randomly select a real, positive point:

$$\begin{aligned}
\ell &= 6.491154749564521\\
x_1 &= 7.317223856586703\\
x_2 &= 6.477459631363067\\
x_3 &= 4.509237064309449\\
k_o &= 5.470088922863450\\
k_c &= 2.963208056077732\\
k_u &= 7.446928070741562\\
k_s^{(2)} &= 1.889550150325445\\
k_s^{(3)} &= 6.867754333653150\\
k_l^{(2)} &= 1.835111557372697\\
k_l^{(3)} &= 3.684845964903365
\end{aligned}$$

where each coordinate is chosen uniformly on the interval $[0, 10]$. This point will determine the observable, i.e. output, variables λ, ρ, and ζ using equations (6)–(8). Call this point $(a^\star, x^\star, z^\star) \in \mathbb{R}^{14}$.

Our aim then is to solve the constrained optimization problem:

$$\begin{aligned}
&\underset{y}{\text{minimize}} && \|(a, x, z) - (a^\star, x^\star, z^\star)\|^2\\
&\text{subject to} && (a, x, z) \in (\mathcal{V}_{\mathcal{M}})_{\mathbb{R}} \cap (\mathcal{V}_{\mathcal{D}})_{\mathbb{R}}.
\end{aligned} \tag{10}$$

Geometrically, we are minimizing the distance between the chosen point $(a^\star, x^\star, z^\star)$ and $(\mathcal{V}_{\mathcal{M}})_{\mathbb{R}} \cap (\mathcal{V}_{\mathcal{D}})_{\mathbb{R}}$.

We will refer to the system defining $(\mathcal{V}_{\mathcal{M}}) \cap (\mathcal{V}_{\mathcal{D}})$ as $f^*(a, x, z)$. Squaring up the polynomial system will be a necessary step when utilizing the perturbed regenerative solving scheme. Squaring up was briefly described in Section 1.4 but we will give additional details here. First notice from previous computations that $\mathcal{V}_{\mathcal{M}} \cap \mathcal{V}_{\mathcal{D}}$ has codimension $14 - 6 = 8$. Thus, there exists a nonempty Zariski open set $\mathcal{A} \subseteq \mathbb{C}^{8\times 9}$ such that for every matrix $A \in \mathcal{A}$, we have $\mathcal{V}_{\mathcal{M}} \cap \mathcal{V}_{\mathcal{D}} \subseteq \mathcal{V}(Af^*(a, x, z))$. In practice, elements of A are chosen uniformly along the complex unit circle. If a point $(a, x, z) \in \mathcal{V}(Af^*(a, x, z))$, then $(a, x, z) \in \mathcal{V}_{\mathcal{M}} \cap \mathcal{V}_{\mathcal{D}}$ may be verified by function evaluation of f^*. If $\mathcal{V}_{\mathcal{M}} \cap \mathcal{V}_{\mathcal{D}}$ is to contain a smooth real point, then $(\mathcal{V}_{\mathcal{M}})_{\mathbb{R}} \cap (\mathcal{V}_{\mathcal{D}})_{\mathbb{R}}$ has real dimension 6.

We will utilize the homotopy developed in [6] find a real point on the component $(\mathcal{V}_{\mathcal{M}})_{\mathbb{R}} \cap (\mathcal{V}_{\mathcal{D}})_{\mathbb{R}}$. The polynomial system that will arise are the so-called Fritz John conditions for optimality. That is, if $(a, x, z) \in (\mathcal{V}_{\mathcal{M}})_{\mathbb{R}} \cap (\mathcal{V}_{\mathcal{D}})_{\mathbb{R}}$ is a critical point of (10) it must satify the Fritz John conditions; a polynomial

system of equations. We would like a homotopy method to find all solutions that satify the Fritz John conditions. In turn, this will find all critical points of (10) which is sufficient enough to find real points on $(\mathcal{V}_{\mathcal{M}})_{\mathbb{R}} \cap (\mathcal{V}_{\mathcal{D}})_{\mathbb{R}}$ and produce positive results on model compatibility. We summarize the homotopy method and Fritz John conditions of optimality for model compatibility of the cell death cluster model.

Given a point $(a^*, x^*, z^*) \in \mathbb{R}^{14}/(\mathcal{V}_{\mathcal{M}})_{\mathbb{R}} \cap (\mathcal{V}_{\mathcal{D}})_{\mathbb{R}}$, the Fritz John condition for optimality states that $\xi = (a, x, z) \in (\mathcal{V}_{\mathcal{M}})_{\mathbb{R}} \cap (\mathcal{V}_{\mathcal{D}})_{\mathbb{R}}$ is a local critical point of $\|(a, x, z) - (a^*, x^*, z^*)\|^2$ if there exists a $\tilde{\lambda} \in \mathbb{P}^8$, complex projective space, so that $(\xi, \tilde{\lambda}) \in (\mathcal{V}_{\mathcal{M}})_{\mathbb{R}} \cap (\mathcal{V}_{\mathcal{D}})_{\mathbb{R}} \times \mathbb{P}^8$ satifies:

$$
\begin{aligned}
Af^* &= 0 && (11)\\
((a, x, y) - (a^*, x^*, z^*))^T \lambda_0 + J(Af^*)^T (\lambda_1, \ldots, \lambda_8)^T &= 0 && (12)
\end{aligned}
$$

where $\lambda = [\lambda_0, \ldots, \lambda_8] \in \mathbb{P}^8$, and $J(Af^*)$ denotes the Jacobian matrix of the functions Af^* w.r.t. (a, x, z). A generic affine patch of $\mathbb{P}^8$ may be chosen that the system may be solved using affine coordinates. That is, there is a nonempty Zariski open subset $\mathcal{B} \in \mathbb{C}^9$ so that for every $\alpha \in \mathcal{B}$ the Fritz John conditions may be solved in affine coordinates:

$$
\begin{aligned}
Af^* &= 0 && (13)\\
((a, x, z) - (a^*, x^*, z^*))^T \lambda_0 + J(Af^*)^T (\lambda_1, \ldots, \lambda_8)^T &= 0 && (14)\\
\alpha_0 \lambda_0 + \alpha_1 \lambda_1 + \cdots \alpha_8 \lambda_8 - 1 &= 0 && (15)
\end{aligned}
$$

In practice, the components of α are chosen uniformly on the complex unit circle. Using the numerical irreducible decomposition, we verified that $\mathcal{V}_M \cap \mathcal{V}_D$ is a complex 6 dimensional algebraic set using witness sets. These conditions satisfy Theorem 5 of [6]. Let $w \in \mathbb{R}^8, \gamma \in \mathbb{C}$, and homotopy $H : \mathbb{C}^{14} \times \mathbb{C}^9 \times \mathbb{C} \to \mathbb{C}^{23}$ be defined by:

$$
H(a, x, y, \lambda, t) = \begin{bmatrix} Af^* - t\gamma w \\ ((a, x, z) - (a^*, x^*, z^*))^T \lambda_0 + J(Af^*)^T (\lambda_1, \ldots, \lambda_8)^T \\ \alpha_0 \lambda_0 + \alpha_1 \lambda_1 + \cdots \alpha_8 \lambda_8 - 1 \end{bmatrix}. \quad (16)
$$

H has the properties that the roots of $H(a, x, z, \lambda, 1)$ are finite and nonsingular, the number of solutions of $H(a, x, z, \lambda, 1) = 0$ is maximal for generically chosen $w, \gamma, (a^*, x^*, z^*), \alpha$, and the one real dimensional solution paths defined by H stating at $t = 1$ allow one to compute the solutions ξ that satisfy the Fritz John condition after projecting solutions $(\xi, \tilde{\lambda}) \to \xi$ as $t \to 0$ along the positive real numbers. The system consists of 23 variables and 23 equations.

Regarding implementation, the nonsingular isolated roots of $H(a, x, z, \lambda, 1)$ are computed numerically using the regeneration [12] method implemented with `Bertini` [3]. Regeneration reduces computation, compared to standard methods such as a total degree homotopy [2], by detecting linear produce structure within the polynomials and when only nonsingular solutions are desired. Theorem 5 of [6] guarantees only nonsingular solutions to $H(a, x, z, \lambda, 1)$ so that regeneration is appropriate. After the roots of $H(a, x, z, \lambda, 1)$ are obtained using regeneration, we use a standard straight-line homotopy, often referred generally to as a parameter homotopy, using (16) implemented in `Bertini` [3] to numerically approximate roots as $t \to 0$. For $t \approx 0$, we approximate critical points by projection onto the variables; $\pi(a, x, z, \lambda) = (a, x, z)$.

Since many of the subexpression used are affine linear we have employed the use of intrinsically defined variables (see Appendix F.1.2 of [2]) to significantly reduce computation. Regeneration and parameter homotopy runs are implemented using $x_1, k_o, k_c, k_u, k_s^{(2)}, k_s^{(3)}, k_l^{(2)}, k_l^{(3)}$. The others, $\ell, x_2, x_3, \lambda, \rho, \zeta$, are parameterized and easily obtained. Input files may be found in the files `Cluster_Step1` and `Cluster_Step2`.

Timing summaries are found in Table 1. Timings include computing the numerical irreducible decomposition of $\mathcal{V}_{\mathcal{M}}$, the numerical irreducible decomposition of $\mathcal{V}_{\mathcal{M}} \cap \mathcal{V}_{\mathcal{D}}$, and the two steps to approximate the critical points. The numerical irreducible decompositions and parameter homotopy were implemented on a Apple MacBook Pro with 2.4 GHz Intel "Core i5" processor using a serial implementation of `Bertini`. Regeneration was implemented on 24 (2.67 GHz Xeon-5650) compute nodes with a CentOS 5.11 OS using a parallel implementation of `Bertini`.

After approximating and examining all critical points, there are three solutions that correspond to real points on $(\mathcal{V}_{\mathcal{M}})_{\mathbb{R}} \cap (\mathcal{V}_{\mathcal{D}})_{\mathbb{R}}$. Among the three real solutions, two solutions are nonnegative. Solutions are

Table 1: Timings collected over 20 runs. The table includes the average time and standard deviations associated to the four computations described in this section.

	Timing
Compute $\mathcal{V}_{\mathcal{M}}$	0.79 sec $\pm$ 0.10 sec
Compute $\mathcal{V}_{\mathcal{M}} \cap \mathcal{V}_{\mathcal{D}}$	0.35 sec $\pm$ 0.10 sec
Regeneration (parallel)	13.69 sec $\pm$ 2.40 sec
Parameter Homotopy	0.04 sec

Table 2: Nonnegative real solutions to $(\mathcal{V}_{\mathcal{M}})_{\mathbb{R}} \cap (\mathcal{V}_{\mathcal{D}})_{\mathbb{R}}$

	Solution 1	Solution 2
ℓ	1.7784308	1.7784308
x_1	0.0545838	0.0141547
x_2	0.0952853	0.1357144
x_3	2.1689611	2.1689611
λ	1.7784308	1.7784308
ρ	2.3188302	2.3188302
ζ	2.1689611	2.1689611
k_o	5.3966315	0.1924532
k_c	3.0914404	3.2734881
k_u	3.9540082	0.2856796
$k_s^{(2)}$	1.9881072	1.2768451
$k_s^{(3)}$	7.6931353	6.9985113
$k_l^{(2)}$	1.9131209	1.8363315
$k_l^{(3)}$	3.6997123	3.6848663

listed in Table 2. We verify these are solutions to $(\mathcal{V}_{\mathcal{M}})_{\mathbb{R}} \cap (\mathcal{V}_{\mathcal{D}})_{\mathbb{R}}$ by function evaluation of $f^*(a,x,z)$ and thus are also solutions of $(\mathcal{V}_{\mathcal{M}})_{\mathbb{R}}$. We conclude from these computations that the clustering model $\mathcal{V}_{\mathcal{M}}$ is compatible with the observable data y.

There are a few natural concerns using this method:

1. We may need to determine model compatibility from sampling a distribution of output variables $z = (\lambda, \rho, \zeta)$. Furthermore, in each case determining model compatibility depends on selecting a real point as input to optimization problem (10).

2. A general model and data variety intersection, $\mathcal{V}_{\mathcal{M}} \cap \mathcal{V}_{\mathcal{D}}$, may be composed of several complex components of varying dimension. Futhermore, each pure-dimensional component may consist of several irreducible components that may either be conjugate paired or self-conjugate. In the later case, real points of $(\mathcal{V}_{\mathcal{M}}) \cap (\mathcal{V}_{\mathcal{D}})$ may be contained on the intersection of these components and whose points are smaller real dimension than expected.

The first case may be addressed by employing a parameter homotopy scheme. In this case, we only need to apply the regeneration method once and may then solve every instance of each problem using an efficient straight-line homotopy on the order of 0.03 sec, as in Table 1. Additional details on parameter homotopies can be found in [1, 2, 13, 14] and large-scale problems may be implemented using `Paramotopy` [15] on the order of millions of parameter values.

The later case may be addressed using the theory from [6], guaranteeing that at least one real point is obtained on each real connected component. Regarding implementation, care must be taken in order to construct a system Af^* so that the codimension of $\mathcal{V}(Af^*)$ pertains to the dimension of each pure-dimensional component $(\mathcal{V}_{\mathcal{M}}) \cap (\mathcal{V}_{\mathcal{D}})$. In the case that their are several pure-dimensional component, one

Table 3: Each reaction described highlights whether the reaction is a forward or reversible reaction by the arrows. Here $i = 1, 2$.

monomer-dimer ($\mathcal{M}_1$)	dimer-dimer ($\mathcal{M}_2$)	single operator ($\mathcal{M}_3$)
$X_i \xrightarrow{k_{basi}} X_i + P_i$	$X_i \xrightarrow{k_{basi}} X_i + P_i$	$X_i \xrightarrow{k_{basi}} X_i + P_i$
$P_i \xrightarrow{k_{degi}} \emptyset$	$P_i \xrightarrow{k_{degi}} \emptyset$	$P_i \xrightarrow{k_{degi}} \emptyset$
$2P_2 \underset{k_{kR}}{\overset{k_{kF}}{\rightleftharpoons}} P_2P_2$	$2P_2 \underset{k_{kR}}{\overset{k_{kF}}{\rightleftharpoons}} P_2P_2$	$2P_2 \underset{k_{kR}}{\overset{k_{kF}}{\rightleftharpoons}} P_2P_2$
$X_1 + P_2P_2 \underset{k_{nR}}{\overset{k_{nF}}{\rightleftharpoons}} X_1P_2P_2$	$X_1 + P_2P_2 \underset{k_{nR}}{\overset{k_{nF}}{\rightleftharpoons}} X_1P_2P_2$	$X_2 + P_2P_2 \underset{k_{qR}}{\overset{k_{qF}}{\rightleftharpoons}} X_2P_2P_2$
$X_2 + P_1 \underset{k_{cR}}{\overset{k_{cF}}{\rightleftharpoons}} X_2P_1$	$X_2 + P_1P_1 \underset{k_{oR}}{\overset{k_{oF}}{\rightleftharpoons}} X_2P_1P_1$	$X_2P_2P_2 \xrightarrow{k_w} X_2P_2P_2 + P_2$
	$2P_1 \underset{k_{\iota R}}{\overset{k_{\iota F}}{\rightleftharpoons}} P_1P_1$	

homotopy must be solved for each dimension present. The clustering model only had one pure-dimensional component of codimension 8, so only one homotopy was required to find all critical points in optimization problem (10).

In summary the steps for model compatibility for the clustering model are as follows:

1. Determine the dimension of the pure-dimensional components of $\mathcal{V}_\mathcal{M} \cap \mathcal{V}_\mathcal{D}$ using the numerical irreducible decomposition.
2. Using the information gathered in step 1, construct equivilent system(s) Af^* where $\mathcal{V}_\mathcal{M} \cap \mathcal{V}_\mathcal{D} \subseteq Af^*$.
3. Using the system(s) in step 2, solve optimization problem (10) by selecting a random real point whose coordinates are sampled uniformly among a nonnegative closed interval and find roots of $H(a, x, z, \lambda, 1)$.
4. Using the roots of $H(a, x, z, \lambda, t)$ at $t = 1$ obtained from step 3, use a straight-line homotopy to approximate solutions (a, x, z, λ) for $t \approx 0$.
5. Obtain critical points from step 4 by projecting $\pi(a, x, z, \lambda) = (a, x, z)$ and filtering real positive solutions.
6. Conclude that the model variety $\mathcal{V}_\mathcal{M}$ is compatible with the data if there are solutions obtained in step 5.

4.2 Synthetic biology and experimental design

We demonstrate an example from synthetic biology with excess intersection ($\dim(\mathcal{V}_\mathcal{M} \cap \mathcal{V}_\mathcal{D}) > 0$). We compare three bistable bio-circuits models analyzed in [16]: monomer-dimer toggle circuit ($\mathcal{M}_1$), dimer-dimer toggle circuit ($\mathcal{M}_2$), and single operator gene circuit ($\mathcal{M}_3$), which were initially presented in [17–19]. The model variables include concentrations of genes (X_i) and proteins (P_i) where $i = 1, 2$ as well as species complexes of the form $X_jP_iP_i, P_iP_i$.

We follow the same notation for variables and parameters as presented by [16]. The reactions governing each of the models are given in Table 3.

These variables interact following mass-action kinetics and form systems of polynomial differential equations where $\mathcal{M}_1, \mathcal{M}_2$, and $\mathcal{M}_3$ have 7, 8 and 6 model variables, respectively, and 10, 12, and 9 kinetic parameters, respectively. The models can be reduced by assuming that the total amount of gene 1 ($X_{1_{tot}}$) and gene 2 ($X_{2_{tot}}$) is conserved and these polynomial systems for each model are as follows. For simplicity, we use P_{11} and P_{22} for P_1P_1 and P_2P_2, respectively.

The monomer-dimer toggle circuit ($\mathcal{M}_1$) system is:

$$\begin{aligned}
-k_{deg1}P_1 - k_{cF}X_2P_1 + k_{bas1}X_1 + k_{cR}(X_{2_{tot}} - X_2) &= 0\\
-2k_{kF}P_2^2 - k_{deg2}P_2 + 2k_{kR}P_{22} + k_{bas2}X_2 &= 0\\
k_{kF}P_2^2 - k_{kR}P_{22} - k_{nF}P_{22}X_1 + k_{nR}(X_{1_{tot}} - X_1) &= 0\\
k_{nR}(X_{1_{tot}} - X_1) - k_{nF}P_{22}X_1 &= 0\\
k_{cR}(X_{2_{tot}} - X_2) - k_{cF}P_1X_2 &= 0.
\end{aligned}$$

The model dimer-dimer toggle circuit ($\mathcal{M}_2$) system is:

$$\begin{aligned}
-2k_{iF}P_1^2 - k_{deg_1}P_1 + 2k_{iR}P_{11} + k_{bas1}X_1 &= 0\\
k_{iF}P_1^2 - k_{iR}P_{11} - k_{oF}P_{11}X_2 + k_{oR}(X_{2_{tot}} - X_2) &= 0\\
-2k_{kF}P_2^2 - k_{deg2}P_2 + 2k_{kR}P_{22} + k_{bas2}X_2 &= 0\\
k_{kF}P_2^2 - k_{kR}P_{22} + k_{nF}P_{22}X_1 + k_{nR}(X_{1_{tot}} - X_1) &= 0\\
k_{nR}(X_{1_{tot}} - X_1) - k_{nF}P_{22}X_1 &= 0\\
k_{oR}(X_{2_{tot}} - X_2) - k_{oF}P_{11}X_2 &= 0.
\end{aligned}$$

The model single-operator positive feedback circuit ($\mathcal{M}_3$) system is:

$$\begin{aligned}
k_{bas2}X_2 - k_{deg2}P_2 - 2k_{kF}P_2^2 + 2k_{kR}P_{22} + k_w(X_{2_{tot}} - X_2) &= 0\\
k_{kF}P_2^2 - k_{kR}P_{22} - k_{qF}P_{22}X_2 + k_{qR}(X_{2_{tot}} - X_2) &= 0\\
k_{qR}(X_{2_{tot}} - X_2) - k_{qF}P_{22}X_2 &= 0
\end{aligned}$$

In this example, we suppose that the total amounts $X_{1_{tot}}$ and $X_{2_{tot}}$ and specific protein synthesis and degradation parameters k_{bas_1}, k_{bas_2}, k_{deg_1}, and k_{deg_2} are known and we assume that our data are measurements of P_1, P_2, and their complexes P_{11}, and P_{22}, i.e. $y = (P_1, P_2, P_{11}, P_{22})$. The aim is to select the best model $\mathcal{M}_1$, $\mathcal{M}_2$, and $\mathcal{M}_3$ given the data. We simulate steady-state data $(P_1, P_2, P_{11}, P_{22}) = (0.4224, 2.4153, 0.9022, 0.4758)$ from the dimer-dimer toggle model ($\mathcal{M}_2$) using the following parameter and variable values:

parameter	value	parameter	value
$X_{1_{tot}}$	1.2099	k_{nF}	1.3566
$X_{2_{tot}}$	2.0660	k_{nR}	0.6521
k_{bas_1}	0.8718	k_{oF}	1.5169
k_{bas_2}	1.6930	k_{oR}	1.0661
k_{deg_1}	1.2550	k_{iF}	3.3169
k_{deg_2}	0.6341	k_{iR}	0.6559
k_{kF}	0.6580	k_{qF}	0.5057
k_{kR}	8.0681	k_{qR}	0.4844
k_{cF}	0.4675	k_w	0.1478
k_{cR}	1.1636		

We add Gaussian noise from $\mathcal{N}(0, 0.1)$ and then find $\dim(\mathcal{V}_{\mathcal{M}_i} \cap \mathcal{V}_{\mathcal{D}})$ for $i = 1, 2, 3$. We can compute the dimension of each intersection using the `dim` command in Macaulay2 or by computing a NID in `Bertini`; we find:

$$\begin{aligned}
\dim(\mathcal{V}_{\mathcal{M}_1} \cap \mathcal{V}_{\mathcal{D}}) &= 3,\\
\dim(\mathcal{V}_{\mathcal{M}_2} \cap \mathcal{V}_{\mathcal{D}}) &= 4,\\
\dim(\mathcal{V}_{\mathcal{M}_3} \cap \mathcal{V}_{\mathcal{D}}) &= 3.
\end{aligned}$$

Some further computations are required to find $\dim((\mathcal{V}_{\mathcal{M}_i})_{\mathbb{R}} \cap (\mathcal{V}_{\mathcal{D}})_{\mathbb{R}}$. Specifically, we need to find real points in each intersection and determine whether or not those points are smooth. Computing the dimension of

the real part of the intersections is more work than necessary for Algorithm 2, however, it provides an illustrative example on how to work with real varieties and the algorithm in [6].

Let $f^{(i)} = 0$ be the polynomial system defining $\mathcal{V}_{\mathcal{M}_i} \cap \mathcal{V}_{\mathcal{D}}$ for $i = 1, 2, 3$ and let $w^{(1)} \in \mathbb{R}^{17}$, $w^{(2)} \in \mathbb{R}^{20}$, $w^{(3)} \in \mathbb{R}^{15}$ be random points. Let $x^{(i)}$ be the vector of indeterminates (unknown parameters and variables) for ith model, and let c_i be the codimension of $\mathcal{V}_{\mathcal{M}_i} \cap \mathcal{V}_{\mathcal{D}}$. We can find a real point on every component of each interesection, by solving the system:

$$f^{(i)} = 0, \tag{17}$$

$$\lambda_1 \nabla f_1^{(i)} + \dots \lambda_{c_i} \nabla f_{c_i}^{(i)} + (x^{(i)} - w^{(i)}) = 0. \tag{18}$$

This is a simplified version of the system in Theorem 5 from [6]. As a remark, notice the similarity of the (17)-(18) to the system in Theorem 1. Algorithms for finding real points on a variety have been built on algorithms for minimizing the distance between a point and a variety since [20].

Once we have a real point on every component of $\mathcal{V}_{\mathcal{M}_i} \cap \mathcal{V}_{\mathcal{D}}$, we can quickly determine $\dim(\mathcal{V}_{\mathcal{M}_i})_{\mathbb{R}} \cap (\mathcal{V}_{\mathcal{D}})_{\mathbb{R}}$ if those real points are smooth. Indeed, if $\mathcal{V}$ is an irreducible variety, then $\dim \mathcal{V} = \dim \mathcal{V}_{\mathbb{R}}$ if $\mathcal{V}$ contains a real smooth point (see [2, §14.1]). Checking whether a real point is smooth can be done by evaluating the Jacobian $\mathcal{V}_{\mathcal{M}_i} \cap \mathcal{V}_{\mathcal{D}}$ at the point; if the Jacobian has full rank, then the point is smooth. In our case, for the three models, every point we find is smooth and thus we are able to reach the conclusion:

$$\dim((\mathcal{V}_{\mathcal{M}_1})_{\mathbb{R}} \cap (\mathcal{V}_{\mathcal{D}})_{\mathbb{R}}) = 3,$$
$$\dim((\mathcal{V}_{\mathcal{M}_2})_{\mathbb{R}} \cap (\mathcal{V}_{\mathcal{D}})_{\mathbb{R}}) = 4,$$
$$\dim((\mathcal{V}_{\mathcal{M}_3})_{\mathbb{R}} \cap (\mathcal{V}_{\mathcal{D}})_{\mathbb{R}}) = 3.$$

The dimension analysis of the varieties $\mathcal{V}_{\mathcal{M}_i} \cap \mathcal{V}_{\mathcal{D}}$ informs us about the minimum number of additional variable and parameter values that must be measured to ensure $\mathcal{V}_{\mathcal{M}} \cap \mathcal{V}_{\mathcal{D}} = \emptyset$. For $\mathcal{M}_1$ we need to know at least 4 more variable and/or parameter values, for $\mathcal{M}_2$ we need to know at least 5 more, and for $\mathcal{M}_3$ we need to know at least 4 more. Thus for the remainder of the example, we assume that we the parameters k_{cF}, k_{cR}, k_{nF}, and k_{kF} are known in $\mathcal{M}_1$, the parameters k_{kF}, k_{nF}, k_{iF}, k_{oF}, and k_{oR} are known in $\mathcal{M}_2$, and the parameters k_{kF}, k_{qF}, k_{qR} and k_w are known in $\mathcal{M}_3$. The model $\mathcal{M}_3$ is an example where the number of additional parameters and/or variables that need to be known/measured exceeds the amount predicted by the dimension analysis.

Now that all the intersections are empty, we run Algorithm 2, using the regeneration methods in Bertini to solve the systems resulting from Theorem 1. We find that the sum of squares (Eq. (3.1)) for each model are as follows: $d_1^2 = 2.116$, $d_2^2 = 0.000124$, and $d_3^2 = 0.6333$. Therefore, we select the $\mathcal{M}_2$ model, which matches the model from which the data was generated.

Solving the zero-dimensional system for the monomer-dimer toggle circuit, $\mathcal{M}_1$, took 1 minute and 3 seconds on an Apple MacBook Pro with a 2.6 GhHz Intel Core i5 processor. Solving the system for the dimer-dimer toggle circuit, $\mathcal{M}_2$, took 1 minute and 43 seconds, and solving the system for the single-operator positive feedback circuit, $\mathcal{M}_3$ took 0.092 seconds.

4.3 Epidemiology HIV

To demonstrate parameter estimation we use a model that includes long-term HIV dynamics from initial virus, latency, and virus increase [21], based on [22]. Model variables x are uninfected CD4+T cells (T), infected CD4$^+$ T cells (T_i), uninfected macrophages (M), infected macrophages (M_i), and HIV virus population (V). The reactions are considered for this model are shown in Table 4.

Table 4: Reactions for HIV model. The published parameter value is used from [21], see references therein.

Description	Reaction	Parameter value
Generation of new CD4+T cells	$\emptyset \xrightarrow{s_1} T$	10
Generation of new macrophages	$\emptyset \xrightarrow{s_2} M$	1.5 $\times$ 10^{-1}
Proliferation of T cells by presence of pathogen	$T + V \xrightarrow{k_1} (T + V) + T$	2 $\times$ 10^{-3}
Infection of T cells by HIV	$T + V \xrightarrow{k_2} T_i$	3 $\times$ 10^{-3}
Proliferation of M by presence of pathogen	$M + V \xrightarrow{k_3} (M + V) + M$	7.45 $\times$ 10^{-4}
Infection of M by HIV	$M + V \xrightarrow{k_4} M_i$	5.22 $\times$ 10^{-4}
Proliferation of HIV within CD4+T cell	$T_i \xrightarrow{k_5} V + T_i$	5.37 $\times$ 10^{-1}
Proliferation of HIV within macrophage	$M_i \xrightarrow{k_6} V + M_i$	2.85 $\times$ 10^{-1}
Natural death of CD4+T cells	$T \xrightarrow{\delta_1} \emptyset$	0.01
Natural death of infected T cells	$T_i \xrightarrow{\delta_2} \emptyset$	0.44
Natural death of macrophages	$M \xrightarrow{\delta_3} \emptyset$	6.6 $\times$ 10^{-3}
Natural death of infected macrophages	$M_i \xrightarrow{\delta_4} \emptyset$	6.6 $\times$ 10^{-3}
Natural death of HIV	$V \xrightarrow{\delta_5} \emptyset$	3

From these reactions, the dynamics are described by the following equations:

$$\begin{aligned}
\dot{T} &= s_1 + k_1 TV - k_2 TV - \delta_1 T \\
\dot{T_i} &= k_2 TV - \delta_2 T_i \\
\dot{M} &= s_2 + k_3 MV - k_4 MV - \delta_3 M \\
\dot{M_i} &= k_4 MV - \delta_4 M_i \\
\dot{V} &= k_5 T_i + k_6 M_i - \delta_5 V
\end{aligned}$$

Using `Macaulay2`, we find that the model variety $\mathcal{V}_\mathcal{M}$ has two irreducible components, the main component $\mathcal{V}_1$ defined by the ideal:

$$\begin{aligned}
&I_1 = \langle 5742M - 2453M_i - 130500, 259908T_i - 46607M_i + 4840000\delta_5 - 20200500, \\
&17721T + 46607M_i - 4840000\delta_5 + 2479500, 484000V\delta_5 - 184547M_i + 4840000\delta_5 - 20200500, \\
&2453M_iV - 72600M_i + 130500V\rangle
\end{aligned}$$

and an extinction component V_2 defined by the ideal:

$$I_2 = \langle V, M_i, 11M - 250, T_i, T - 1000\rangle$$

We estimated the natural death of the virus, parameter δ_5, using Algorithm 3 with main component $\mathcal{V}_1$ in place of $\mathcal{V}_\mathcal{M}$ (see Section 3.2). For $\mathcal{V}_\mathcal{D}$, we used the long-term non-progressors steady-state value (Table 3 of [21]) and added noise to each variable $\sim \mathcal{N}(0, 1)$. In particular, the data variety $\mathcal{V}_\mathcal{D}$ is defined by the

equations:

$$T - \frac{6383}{20} = 0$$
$$T_i - \frac{937}{20} = 0$$
$$M - \frac{8109}{100} = 0$$
$$M_i - \frac{13667}{100} = 0$$
$$V - \frac{2121}{100} = 0$$

For $s_1, s_2, k_1, \dots, k_6, \delta_1, \dots, \delta_4$, we treated these parameters as known using the values from Table 1 of [21].

The varieties $\mathcal{V}_1$ and $\mathcal{V}_{\mathcal{D}}$ do not intersect, which we can confirm with `Macaulay2`. Using `Bertini`, we solve the following system from Proposition 1 in the main text:

$$5742M - 2453M_i - 130500 = 0$$
$$259908T_i - 46607M_i + 4840000\delta_5 - 20200500 = 0$$
$$17721T + 46607M_i - 4840000\delta_5 + 2479500, 484000V\delta_5 - 184547M_i + 4840000\delta_5 - 20200500 = 0$$
$$2453M_iV - 72600M_i + 130500V = 0$$
$$T + 17721\lambda_3 - 6383/20 = 0$$
$$T_i + 259908\lambda_2 - 937/20 = 0$$
$$M + 5742\lambda_1 - 8109/100 = 0$$
$$2453\lambda_5 + M_i - 2453\lambda_1 - 46607\lambda_2 + 46607\lambda_3 - 184547\lambda_4 - 72600\lambda_5 - 13667/100 = 0$$
$$484000\delta_5\lambda_4 + 2453M_i\lambda_5 + V + 130500\lambda_5 - 2121/100 = 0$$
$$484000V\lambda_4 + 4840000\lambda_2 - 4840000\lambda_3 + 4840000\lambda_4 = 0$$

There are 16 complex solutions to this equation, 3 of which are real. The real point resulting in the smallest sum of squared errors $d^2 = 0.2884$ is:

$$(T, T_i, M, M_i, V, \delta_5, \lambda_1, \lambda_2, \lambda_3, \lambda_4, \lambda_5) =$$
$$(319.408, 46.404, 81.1544, 136.767, 21.3079, 2.99876,$$
$$-0.0000112074, 0.00000171594, -0.0000145481, -0.00000519486, 0.0000159701)$$

Thus, Algorithm 3 returns $\bar{\delta}_5 = 2.99876$, which we can compare to the true value $\delta_5 = 3$. Solving the system on an Apple MacBook Pro with a 2.6 GhHz Intel Core i5 processor took 48 seconds.

4.4 Multisite phosphorylation

Here we describe the details for the multisite phosphorylation model selection and parameter estimation computations. First we describe the relevant biology, next we present the mathematical models of the distributive and processive mechanisms, then we apply our model selection method using data from [23]. We also estimate the relationship between the EGF concentration and activation of the pathway described by the parameter k_1 (see Table 11).

4.4.1 Biology of MAP Kinase system

Many cellular decisions are governed by molecular post-translational modifications. One type of modification, phosphorylation, is the addition of a phosphate group to a site of a substrate by an enzyme called a kinase. Some proteins (substrates) require multiple phosphate groups to be added by the kinase before the protein in activated/de-activated by these modifications. One well-studied signaling system is the MAP Kinase pathway, with kinase MEK and its substrate ERK; however the mechanism by which the phosphate

Table 5: Description of variables and parameters for distributive and processive MAP Kinase models

variable	species	parameter	name	parameter	name
x_1	MEK	k_1	kphos_MEK_pMEK	k_{15}	kdphos_pY_np_cyt
x_2	cRAF	k_2	kdphos_pMEK_MEK	k_{16}	kdphos_pT_np_cyt
x_3	pMEK	k_3	kf_MEK_ERK_binding	k_{17}	kdphos_pTpY_pY_nuc
x_4	np-ERK_cyt	k_4	kb_MEK_ERK_dissociation	k_{18}	kdphos_pTpY_pT_nuc
x_5	MEK_np-ERK	k_5	kimport_np	k_{19}	kdphos_pY_np_nuc
x_6	np-ERK_nuc	k_6	kexport_np	k_{20}	kdphos_pT_np_nuc
x_7	pY-ERK_cyt	k_7	kimport_pY	k_{21}	kphos_np_pY
x_8	pY-ERK_nuc	k_8	kexport_pY	k_{22}	kphos_pY_pTpY
x_9	pT-ERK_cyt	k_9	kimport_pT	k_{23}	kphos_pT_pTpY
x_{10}	pT-ERK_nuc	k_{10}	kexport_pT	k_{24}	kf_MEK_ERK_binding
x_{11}	pTpY-ERK_cyt	k_{11}	kimport_pTpY	k_{25}	kb_MEK_ERK_dissociation
x_{12}	pTpY-ERK_nuc	k_{12}	kexport_pTpY	k_{26}	kphos_np_pY
x_{13}	pMEK_np-ERK	k_{13}	kdphos_pTpY_pY_cyt	k_{27}	kphos_pY_pTpY_MEKERK
x_{14}	pMEK_pY-ERK	k_{14}	kdphos_pTpY_pT_cyt	c_2,c_1	cyt_vol, nuc_vol

group is added has been debated. Either MEK could phosphorylate ERK, disassociate and then phosphorylate again, called *distributive*; or MEK could bind and phosphorylate in sequence, called *processive.* Aoki et al [23] showed experimentally (with mathematical models) that this mechanism is different *in vitro* than *in vivo.* This experiment included 12 different levels of EGF stimulus ranging from 0.0244140625 ng/mL to 50 ng/ML. EGF actives cRAF which then phosphorylates MEK and finally doubly phosphorylates ERK. The data are measurements of three replicates of nonphosphorylated ERK (np-ERK), tyrosine monophosphorylated ERK (pY-ERK), and doubly phosphorylated ERK (pTpY-ERK), at each stimulus level. These data are given as percentage of total ERK (ERK), so we use the concentration measurement for each of these ERK states.

4.4.2 Mathematical models

The model variables and parameters are given in Table 5. The model parameters for the distributive model are:

$$a = (k_1, \dots, k_{27}, c_1, c_2),$$

the variables are:

$$x = (x_1, \dots, x_{12}, \text{cRAF}_{tot}, \text{MEK}_{tot}, \text{ERK}_{tot}),$$

and the outputs are:

$$z = (\text{np-ERK}, \text{pY-ERK}, \text{pYpT-ERK}).$$

The variables for the processive model are the same as for the distributive model except for two additional variables x_{13}, x_{14}. The reaction velocities are given in Table 6 and the corresponding equations are given in Table 7. Note in Table 7 that there are various conserved species concentrations in addition to the ODEs.

We use the *in vitro* parameters estimates from Table S2 in reference [23] for $k_2, \dots, k_{27}, c_1, c_2$ and the conserved quantities MEK_{tot}, cRAF_{tot}, ERK_{tot}, as given in Table 8. The remaining parameter, k_1, describes the rate of MEK phosphorylation and depends on the level of EGF stimulation, which varies throughout the data. The output variables are np-ERK, pY-ERK, and pYpT-ERK, which are sums of species concentrations. For the distributive model, the output equations are:

$$\text{np-ERK} - (x_4 + x_5 + x_6) = 0 \quad (19)$$

$$\text{pY-ERK} - (x_7 + x_8) = 0 \quad (20)$$

$$\text{pYpT-ERK} - (x_{11} + x_{12}) = 0 \quad (21)$$

Table 6: Reaction velocities for the MAP Kinase distributive and processive model. The processive model uses the additional reaction velocities v_{18}, v_{19}, v_{20}.

$v_1 = k_1x_1x_2 - k_2x_3$	$v_2 = k_3x_1x_4 - k_4x_5$	$v_3 = k_5x_4 - c_2k_6x_6$
$v_4 = k_7x_7 - c_2k_8x_8$	$v_5 = k_9x_9 - c_2k_{10}x_{10}$	$v_6 = k_{11}x_{11} - c_2k_{12}x_{12}$
$v_7 = k_{13}x_{11}$	$v_8 = k_{14}x_{11}$	$v_9 = k_{15}x_7$
$v_{10} = k_{16}x_9$	$v_{11} = c_2k_{17}x_{12}$	$v_{12} = c_2k_{18}x_{12}$
$v_{13} = c_2k_{19}x_8$	$v_{14} = c_2k_{20}x_{10}$	$v_{15} = k_{21}x_3x_4$
$v_{16} = k_{22}x_3x_7$	$v_{17} = k_{23}x_3x_9$	
$v_{18} = k_{24}x_3x_4 - k_{25}x_{13}$	$v_{19} = k_{26}x_{13}$	$v_{20} = k_{27}x_{14}$

Table 7: Equations for distributive and processive MAP Kinase models

Variable		Distributive	Processive
$\dot{x}_1$	=	$-v_1 - v_2$	$-v_1 - v_2$
$\dot{x}_2$	=	0	0
$\dot{x}_3$	=	v_1	$v_1 - v_{18} + v_{20}$
$\dot{x}_4$	=	$-v_2 - v_3 + v_9 + v_{10} - v_{15}$	$-v_2 - v_3 + v_9 + v_{10} - v_{18}$
$\dot{x}_5$	=	v_2	v_2
$\dot{x}_6$	=	$v_3 + v_{13} + v_{14}$	$v_3 + v_{13} + v_{14}$
$\dot{x}_7$	=	$-v_4 + v_7 - v_9 + v_{15} - v_{16}$	$-v_4 + v_7 - v_9 - v_{16}$
$\dot{x}_8$	=	$v_4 + v_{11} - v_{13}$	$v_4 + v_{11} - v_{13}$
$\dot{x}_9$	=	$-v_5 + v_8 - v_{10} - v_{17}$	$-v_5 + v_8 - v_{10} - v_{17}$
$\dot{x}_{10}$	=	$v_5 + v_{12} - v_{14}$	$v_5 + v_{12} - v_{14}$
$\dot{x}_{11}$	=	$-v_6 - v_7 - v_8 + v_{16} + v_{17}$	$-v_6 - v_7 - v_8 + v_{16} + v_{17} + v_{20}$
$\dot{x}_{12}$	=	$v_6 - v_{11} - v_{12}$	$v_6 - v_{11} - v_{12}$
$\dot{x}_{13}$	=		$v_{18} - v_{19}$
$\dot{x}_{14}$	=		$v_{19} - v_{20}$
0	=	$\text{MEK}_{tot} - (x_1 + x_3 + x_5)$	$\text{MEK}_{tot} - (x_1 + x_3 + x_5 + x_{13} + x_{14})$
0	=	$\text{cRAF}_{tot} - x_2$	$\text{cRAF}_{tot} - x_2$
0	=	$\text{ERK}_{tot} - \sum_{i=4}^{12} x_i$	$\text{ERK}_{tot} - \sum_{i=4}^{14} x_i$

Table 8: Parameter values for MAP Kinase models

parameter	value	parameter	value	parameter	value
k_2	0.0096	k_{13}	0.004	k_{24}	0.18
k_3	0.18	k_{14}	0.0055	k_{25}	0.27
k_4	0.27	k_{15}	0.0067	k_{26}	0.073
k_5	0.0017	k_{16}	0.0068	k_{27}	0.05
k_6	0.013	k_{17}	0.0032	c_1	1.0
k_7	0.0025	k_{18}	0.0038	c_2	0.2
k_8	0.017	k_{19}	0.0077	cRAF_{tot}	0.013
k_9	0.0022	k_{20}	0.0058	MEK_{tot}	1.2
k_{10}	0.049	k_{21}	0.039	ERK_{tot}	0.74
k_{11}	0.0082	k_{22}	0.021		
k_{12}	0.0076	k_{23}	0.02		

whereas for the processive model, we include two additional species, so the output equations become:

$$\text{np-ERK} - (x_4 + x_5 + x_6 + x_{13}) = 0 \quad (22)$$
$$\text{pY-ERK} - (x_7 + x_8 + x_{14}) = 0 \quad (23)$$
$$\text{pYpT-ERK} - (x_{11} + x_{12}) = 0. \quad (24)$$

4.4.3 Model selection and parameter estimation computations

The model variety $\mathcal{V}_{\mathcal{M}_d}$ of the distributive model is defined by (19) - (21) and the equations obtained by setting the "Distributive" column of Table 7 equal to zero. We will refer to the system defining $\mathcal{V}_{\mathcal{M}_d}$ as F. The model variety $\mathcal{V}_{\mathcal{M}_p}$ of the processive model is defined by (22) - (24) and the equations obtained by setting the "Processive" column of Table 7 equal to zero. The ambient dimension of $\mathcal{V}_{\mathcal{M}_d}$ is 16 since the coordinates that define $\mathcal{V}_{\mathcal{M}_d}$ include $x_1, \ldots, x_{12}$, np-ERK, pY-ERK, pYpT-ERK, and the model parameter k_1; all other parameters and variables we treat as known constants. Similarly the ambient dimension for the processive model is 18 as we include the additional variables x_{13} and x_{14}.

Given data $y = (\text{np-ERK}', \text{pY-ERK}', \text{pYpT-ERK}')$ we define the data variety for the distributive model as:

$$\mathcal{V}_{\mathcal{D}_d} = \left\{(a, x, z) \in \mathbb{C}^{16} : z = y\right\}.$$

The data variety $\mathcal{V}_{\mathcal{D}_d}$ has dimension 13. The data used is found in the supplementary data file (`aoki_data.txt`). The data variety $\mathcal{V}_{\mathcal{D}_p}$ for the processive model is defined similarly. The computations that follow will be for the distributive model. The computations for the processive model will be nearly identical so we do not describe them in the same level of detail. We record information for both models.

We first compute a numerical irreducible decomposition (NID) of $\mathcal{V}_{\mathcal{M}_d}$ using `Bertini` [3]. With the NID, one can verify that $\mathcal{V}_{\mathcal{M}_d}$ is a one-dimensional complex algebraic set of degree 8 (filename: `MAPK_D_Model_NID`). Similarly, for the processive model, the model variety $\mathcal{V}_{\mathcal{M}_p}$ is a one-dimensional complex algebraic set of degree 11 (filename: `MAPK_P_Model_NID`). There are several variables that may be intrinsically defined to save computation. For example, x_1, x_2, x_7, and x_{11} can be written in terms of the other variables followed by x_4. One may also verify that $\mathcal{V}_{\mathcal{M}_d} \cap \mathcal{V}_{\mathcal{D}_d} = \emptyset$ and $\mathcal{V}_{\mathcal{M}_p} \cap \mathcal{V}_{\mathcal{D}_p} = \emptyset$ using `Bertini` (filename(s): `MAPK_D_Model_Data_NID` and `MAPK_P_Model_Data_NID`). We define the variables np-ERK, pY-ERK, and pYpT-ERK intrinsically to save computation. Since $\mathcal{V}_{\mathcal{M}_d} \cap \mathcal{V}_{\mathcal{D}_d} = \emptyset$ and $\mathcal{V}_{\mathcal{M}_p} \cap \mathcal{V}_{\mathcal{D}_p} = \emptyset$, Algorithm 2 instructs us to minimize the distance between $(\mathcal{V}_{\mathcal{M}_d})_{\mathbb{R}}$ and $(\mathcal{V}_{\mathcal{D}_d})_{\mathbb{R}}$ and similiarly between $(\mathcal{V}_{\mathcal{M}_p})_{\mathbb{R}}$ and $(\mathcal{V}_{\mathcal{D}_p})_{\mathbb{R}}$ for each data point.

Squaring up the polynomial system defining $V_{\mathcal{M}_d}$ will be a necessary step to construct the polynomial system from Proposition 1. This procedure was described briefly in Section 1.4 and in more detail in Section 4.1. The codimension of $V_{\mathcal{M}_d}$ is $c = 16 - 1 = 15$, the dimension of the ambient space minus the dimension of $V_{\mathcal{M}_d}$ as determined by the NID. Let $A \in \mathbb{C}^{15\times 17}$ whose entries are taken randomly from the complex unit circle and set $f^*(a, x, z) = AF(a, x, z)$. Our aim is to solve the optimization problem:

$$\begin{array}{ll} \text{minimize} & \|z - y\| \\ \text{subject to} & (a, x, z) \in (\mathcal{V}_{\mathcal{M}})_{\mathbb{R}} \cap \mathbb{R}^{16}_{\geq 0}. \end{array} \quad (25)$$

We set up the Fritz John conditions:

$$f^*(a, x, z) = 0 \quad (26)$$
$$\Sigma_{j=1}^{15} \frac{\partial f_j^*(a, x, z)}{\partial a_1} \lambda_j = 0, \quad (27)$$
$$\Sigma_{j=1}^{15} \frac{\partial f_j^*(a, x, z)}{\partial x_i} \lambda_j = 0, \text{ for } 1 \leq i \leq 12 \quad (28)$$
$$(z_i - y_i)\lambda_0 + \Sigma_{j=1}^{15} \frac{\partial f_j^*(a, x, z)}{\partial z_i} \lambda_j = 0, \text{ for } 1 \leq i \leq 3. \quad (29)$$

where $\lambda = [\lambda_0, \lambda_1, \cdots, \lambda_{15}] \in \mathbb{P}^{15}$. This is a system of 31 variables and 31 equations on $\mathbb{C}^{16} \times \mathbb{P}^{15}$. There exists a nonempty Zariski open subset $\mathcal{A} \subset \mathbb{C}^{16}$ so that for each $\alpha = (\alpha_0, \alpha_1, \ldots, \alpha_{15}) \in \mathcal{A}$, equations

(26)–(29) may be defined using affine coordinates on a patch equation:

$$\alpha_0\lambda_0 + \alpha_1\lambda_1 + \cdots + \alpha_{15}\lambda_{15} - 1 = 0.$$

The Fritz John conditions for optimality state that $(\overline{a}, \overline{x}, \overline{z})$ is a critical point of $\|z-y\|$ for $(a, x, z) \in (\mathcal{V}_{\mathcal{M}})_{\mathbb{R}}$ if there is a $\overline{\lambda} \in \mathbb{P}^{15}$ so that $(\overline{a}, \overline{x}, \overline{z}, \overline{\lambda})$ is a solution to equations (26)–(29). We obtain the critical points by projecting onto (a, x, z); $\pi(a, x, z, \lambda) = (a, x, z)$. Our approach computes every critical point from equations (26)–(29), and evaluates $\|z - y\|$ for each critical point.

In Section 3.1, we explained an issue that can arise in solving constrained optimization problems such as ones arising from equations (1)–(5). In this example, we want to ensure that $x_1, \ldots, x_{12}, a_1, z_1, z_2, z_3$ are non-negative, i.e. $\mathcal{S}_{\mathcal{M}_d} = \mathcal{V}_{\mathcal{M}_d} \cap \mathbb{R}^{16}_{\geq 0}$. To minimize the distance between $\mathcal{S}_{\mathcal{M}_d}$ and $\mathcal{V}_{\mathcal{D}_d}$ using a NAG approach, we solve the system (26)–(29), and then solve the system many more times, setting combinations of $x_1, \ldots, x_{12}, a_1, z_1, z_2, z_3$ to zero. Since the complex dimension of $\mathcal{V}_{\mathcal{M}_d}$ is one, and $\mathcal{V}_{\mathcal{M}_d}$ is does not contain any coordinate hyperplanes as components (i.e. where any coordinates of (a, x, z) are zero), one can verify that $\mathcal{V}_{\mathcal{M}_d}$ restricted to every coordinate hyperplane is zero-dimensional. In this case, checking the various boundary conditions of $\mathcal{V}_{\mathcal{M}_d} \cap \mathbb{R}^{16}_{\geq 0}$ becomes trivial. See Section 3.1 for a more general discussion of this approach.

We consider 36 data points where each data point consists of a triple $y = (\text{np-ERK}', \text{pY-ERK}', \text{pYpT-ERK}')$ define in the supplementary data file (`aoki_data.txt`). Rather than solve equations (26)–(29) independently for each data point, we first define a homotopy $H : \mathbb{C}^{32} \times \mathbb{C}^3 \to \mathbb{C}^{32}$:

$$H(a, x, z, \lambda; p) = \begin{bmatrix} f^*(a, x, z) \\ \Sigma_{j=1}^{15} \frac{\partial f_j^*(a,x,z)}{\partial a_1} \lambda_j \\ \Sigma_{j=1}^{15} \frac{\partial f_j^*(a,x,z)}{\partial x_i} \lambda_j \\ (z_i - p_i)\lambda_0 + \Sigma_{j=1}^{15} \frac{\partial f_j^*(a,x,z)}{\partial z_i} \lambda_j \\ \alpha_0\lambda_0 + \alpha_1\lambda_1 + \cdots + \alpha_{15}\lambda_{15} - 1 \end{bmatrix} \tag{30}$$

using a general parameter $p = (p_1, p_2, p_3) \in \mathbb{C}^3$. When the parameter is specialized to $p = y$, we recover the Fritz John conditions for a given optimization problem using the data y. Using theory of parameter homotopies, there is a nonempty Zariski open subset $\mathcal{P} \subseteq \mathbb{C}^3$ so that nonsingular isolated roots of $H(a, x, z, \lambda; p)$ is maximal as they vary in $\mathcal{P}$. Furthermore, for any $p^* \in \mathcal{P}$, every isolated root of $H(a, x, z, \lambda; y)$ may be obtained by constructing the straight-line homotopy $H(a, x, z, \lambda; p^*t + (1 - t)y)$ and tracking the one real-dimensional solution paths starting at the nonsingular isolated roots of $H(a, x, z, \lambda; p^*)$ and obtaining the isolated roots of $H(a, x, z, \lambda; y)$ as $t \to 0$. Additional details on parameter homotopies can be found in [1, 2, 13, 14] and large-scale problems may be implemented using `Paramotopy` [15] on the order of millions of parameter values.

The benefit of employing a parameter homotopy is immense. Parameter homotopies significantly reduce the computation required since solving the parameterized system at a generic parameter $p = p^*$ only needs to be solved once. Any further specialization of the parameter $p = y$ requires only a fraction of the amount of computation. Concretely, this amounts to around a $58\times$ speed up for the distributive model and approximately a $100\times$ speed up for the processive model.

In addition to employing a parameter homotopy solving scheme, equations (26)–(29), have a natural homogeneous product structure. That is, after equations (26)–(29) are multihomogenized with respect to the product of projective spaces $\mathbb{P}^{16} \times \mathbb{P}^{15}$, where the first space corresponds to the coordinates (a, x, z) and the second space corresponds to the coordinates λ, the number of nonsingular solutions is significantly reduced when compared to the space $\mathbb{P}^{31}$. In unison with parameter homotopies a more efficient homotopy is used to solve $H(a, x, z, \lambda; p^*)$ for $p^* \in \mathcal{P}$. References [1] and [2] contain further details on multihomogeneous homotopies.

One additional reformulation that we can do to reduce computation is to define some of the variables that occur in (26)–(29) intrinsically. This is most easily applied when one or more variables can be expressed as a linear combination of other variables. Specifically, we know from Table 7 that:

$$x_2 = \text{cRAF}_{tot}$$

Table 9: Path counts on processive and distributive models. '$\{(a,x,z),\lambda\}$-hom' corresponds to a $\{(a,x,z),\lambda\}$-homogeneous variable grouping and 'intrinsic x_2' corresponds to the system where x_2 is intrinstically defined.

	Total Degree	$\{(a,x,z),\lambda\}$-hom	$\{(a,x,z),\lambda\}$-hom + intrinsic x_2
Distributive Model	124,416 paths	3,744 paths	1,152 paths
Processive Model	248,832 paths	7,488 paths	2,304 paths

Table 10: Expected timings for the MAPK model collected over 20 'random' runs.

	Compute Dimension	Initial Solve (parallel)	Data Solve (all 36)
Distributive Model	4.50 sec ± 0.53 sec	44.80 sec ± 4.85 sec	27.80 sec ± 3.16 sec
Processive Model	6.64 sec ± 0.48 sec	91.67 sec ± 7.69 sec	32.77 sec ± 5.06 sec

where cRAF_{tot} is defined as a constant in Table 8. Thus, we can "remove" x_2 from our computations. Partial derivatives are no longer necessary with respect to the variable x_2, and x_2 is no longer defined explicitly when tracking homotopy paths.

Table 9 summarizes the sequence of reductions made in the number of paths by imposing a $\{(a,x,z),\lambda\}$-homogeneous structure followed by intrinsically defining the variable x_2 along with the number of paths required using the standard total degree homotopy [1],[2].

Table 12 and Table 13 record the distances between the data and model varieties for all 36 data points. A missing "interior" distance in Table 12 and Table 13 indicate there were no positive real critical points found on the interior of $\mathcal{V}_{\mathcal{M}_d} \cap \mathbb{R}^{16}_{\geq 0}$ for the given EGF level and replicate, for example. However, we may still compute a distance to the boundary of the semi-algebraic set corresponding to each model. `Bertini` input files, shell scripts, and `MATLAB` scripts are available within the supplementary files to analyze model selection and parameter estimation. The distances are summarized graphically in Figure S1.

Timing summaries for both the processive and distributive model can be found in Table 10. These timings include the NID required to compute the dimension of each pure-dimensional component of the model variety $\mathcal{V}_{\mathcal{M}_d}$ and $\mathcal{V}_{\mathcal{M}_p}$, computing the nonsingular solutions of (30) for the distributive model at a generic parameter $p^* \in \mathcal{P}$ required to employ a parameter homotopy scheme (and a similiar solve for the processive model), and the parameter homotopy to solve equations (26)–(29) for each data point (and a similiar parameter homotopy for the processive model). Timings to compute the dimension of the model variety and the data solve were done in serial using a Apple MacBook Pro with 2.4 GHz Intel "Core i5" processor. The initial solves for the parameter homotopies were done in parallel using 96 (2.67 GHz Xeon-5650) compute nodes on the CentOS 5.11 operating system. The data solves were then done in serial using the same MacBook Pro.

References

[1] Sommese AJ, Wampler CW (2005) *The numerical solution to systems of polynomials arising in engineering and science* (World Scientific, Singapore).

[2] Bates DJ, Hauenstein JD, Sommese AJ, Wampler CW (2013) *Numerically solving polynomial systems with Bertini* (SIAM) Vol. 25.

[3] Bates DJ, Hauenstein JD, Sommese AJ, Wampler CW (2014) Bertini: Software for numerical algebraic geometry. available at `bertini.nd.edu`.

Figure S1: Distance Plot

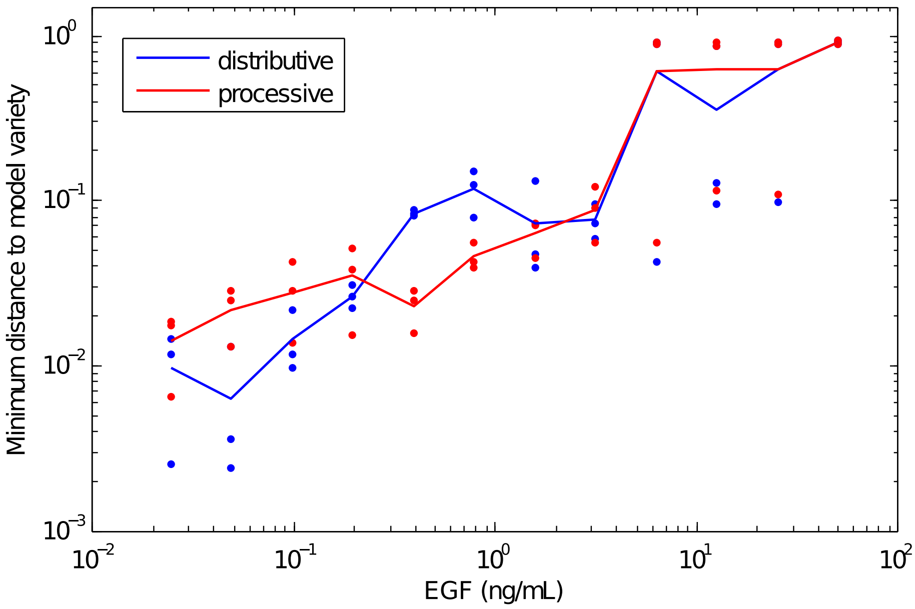

[4] Wu W, Reid G (2013) Finding points on real solution components and applications to differential polynomial systems. *ISSAC '13 Proceedings of the 38th International Symposium on Symbolic and Algebraic Computation* 16:339–346.

[5] Rouillier F, Roy MF, Din MSE (2000) Finding at least one point in each connected component of a real algebraic set defined by a single equation. *Journal of Complexity* 16:716–750.

[6] Hauenstein JD (2013) Numerically computing real points on algebraic sets. *Acta Applicandae Mathematicae* 125:105–119.

[7] Verschelde J (1999) Algorithm 795: Phcpack: A general-purpose solver for polynomial systems by homotopy continuation. *ACM Transactions on Mathematical Software (TOMS)* 25:251–276.

[8] Lee TL, Li TY, Tsai CH (2008) Hom4ps-2.0: a software package for solving polynomial systems by the polyhedral homotopy continuation method. *Computing* 83:109–133.

[9] Rostalski P, Fotiou I, Bates D, Beccuti A, Morari M (2011) Numerical algebraic geometry for optimal control applications. *SIAM Journal on Control and Optimization* 21:417–437.

[10] Gross E, Harrington HA, Rosen Z, Sturmfels B (2016) Algebraic systems biology: A case study for the wnt pathway. *Bulletin of Mathematical Biology* 78:21–51.

[11] Scott FL, et al. (2009 Feb 19) The fas-fadd death domain complex structure unravels signalling by receptor clustering. *Nature* 457:1019–1022.

[12] Hauenstein JD, Sommese AJ, Wampler CW (2011) Regeneration homotopies for solving systems of polynomials. 80:345–377.

[13] Li TY, Sauer T, Yorke JA (1989) The cheater's homotopy: An efficient procedure for solving systems of polynomial equations. *SIAM Journal on Numerical Analysis* 26:1241–1251.

[14] Morgan AP, Sommese AJ (1989) Coefficient-parameter polynomial continuation. *Appl. Math. Comput.* 29:123–160.

[15] Bates DJ, Brake DA, Niemerg ME (2012) Paramotopy: Parameter homotopies in parallel.

[16] Siegal-Gaskins D, Franco E, Zhou T, Murray RM (2014) An analytical approach to bistable biological circuit discrimination using real algebraic geometry. *BioRxiv.*

[17] Gardner T, Cantor C, JJ C (2000) Construction of a genetic toggle switch in Escherichia coli. *Nature* 403:339–342.

[18] Hasty J, Isaacs F, Dolnik M, McMillen D, Collina J (2001) Designer gene networks: Towards fundamental cellular control. *Chaos* 11:207–220.

[19] Siegal-Gaskins D, Mejia-Guerra M, Smith G, E G (2011) Emergence of switch-like behavior in a large family of simple biochemical networks. *PLoS Computational Biology* 7:e1002039.

[20] Seidenberg A (1954) A new decision method for elementary algebra. *Ann. of Math.* 2:365–374.

[21] Hernandez-Vargas EA, Middleton R, Mehta D (2011) Towards modeling hiv long term behavior. *Proceedings of the 18th IFAC World Congress* 18:581–586.

[22] Hadjiandreou M, Conejeros R, Vassiliadis V (2007) Towards a long-term model construction for the dynamic simulation of hiv infection. *Mathematical Bioscience and Engineering* 4:489–504.

[23] Aoki K, Yamada M, Kunida K, Yasuda S, Matsuda M (2011) Processive phosphorylation of ERK MAP kinase in mammalian cells. *Proceedings of the National Academy of Sciences of the United States of America* 108:12675–12680.

Table 11: Parameter estimate of k_1 for distributive and processive MAP Kinase models

EGF level	Distributive	Processive
1 (0.0244140625 ng/mL)	0.006655185015565	0.002630893498836
1	0.005169208080986	0.002666996926269
1	0.010517845922915	0.004869916688582
2 (0.048828125 ng/mL)	0.010599752816972	0.004139244229282
2	0.005294859090858	0.002185712163549
2	0.012645415710607	0.005598240936340
3 (0.09765625 ng/mL)	0.013040547423471	0.005555450037128
3	0.007862190037618	0.003676690633724
3	0.024444604812656	0.010890090940375
4 (0.1953125 ng/mL)	0.022314241866218	0.010026925643431
4	0.014767039426565	0.007566455646162
4	0.032112677267834	0.014358973830329
5 (0.390625 ng/mL)	0.057037089355902	0.028188983627260
5	0.034598433900389	0.018615955020805
5	0.046993978170047	0.023610675947125
6 (0.78125 ng/mL)	0.171132616846835	0.081810937526556
6	0.108600436914435	0.052541291660911
6	0.128469450822610	0.062127115025642
7 (1.5625 ng/ML)	0.552602449693311	0.311829951745706
7	0.198177806441867	0.094130793284515
7	0.307630980846652	0.162410456627762
8 (3.125 ng/ML)	1.535918937663103	1.104298831092815
8	1.558792683503445	0.653311235583313
8	1.114642498639134	0.700052847271073
9 (6.25 ng/ML)	0	0
9	6.741089632274929	2.682148283074402
9	0	0
10 (12.5 ng/ML)	62.84803495830790	0
10	0	0
10	2.556601780466427	1.856045810178470
11 (25 ng/ML)	0	0
11	0	0
11	18.13598179566577	12.162040343795182
12 (50 ng/ML)	0	0
12	0	0
12	0	0

Table 12: Distance to (smaller) distributive model variety

EGF level	Replicate	"Interior" distance	"Boundary" distance
1	1	0.0025	0.0309
	2	0.0118	0.0266
	3	0.0145	0.0496
2	1	0.0024	0.0485
	2	0.0036	0.0249
	3	0.0130	0.0581
4	1	0.0098	0.0594
	2	0.0117	0.0377
	3	0.0218	0.1062
8	1	0.0221	0.0981
	2	0.0312	0.0714
	3	0.0259	0.1349
16	1	0.0870	0.2189
	2	0.0838	0.1559
	3	0.0814	0.1904
32	1	0.1243	0.4343
	2	0.1505	0.3374
	3	0.0791	0.3784
64	1	0.0388	0.6990
	2	0.1312	0.4648
	3	0.0473	0.5889
128	1	0.0959	0.8398
	2	0.0725	0.7501
	3	0.0594	0.7931
256	1	—	0.9093
	2	0.0427	0.8353
	3	—	0.8839
512	1	0.1291	0.9154
	2	—	0.8556
	3	0.0947	0.8597
1024	1	—	0.9111
	2	—	0.8817
	3	0.0970	0.8883
2048	1	—	0.9272
	2	—	0.8948
	3	—	0.9197

Table 13: Distance to (larger) processive model variety

EGF level	Replicate	"Interior" distance	"Boundary" distance
1	1	0.0176	0.0309
	2	0.0066	0.0266
	3	0.0183	0.0496
2	1	0.0281	0.0485
	2	0.0130	0.0249
	3	0.0247	0.0581
4	1	0.0282	0.0594
	2	0.0137	0.0377
	3	0.0421	0.1062
8	1	0.0379	0.0981
	2	0.0154	0.0714
	3	0.0514	0.1349
16	1	0.0284	0.2189
	2	0.0156	0.1559
	3	0.0246	0.1904
32	1	0.0392	0.4343
	2	0.0424	0.3374
	3	0.0561	0.3784
64	1	0.0735	0.6990
	2	0.0444	0.4648
	3	0.0717	0.5889
128	1	0.1218	0.8398
	2	0.0550	0.7501
	3	0.0899	0.7931
256	1	—	0.9093
	2	0.0557	0.8353
	3	—	0.8839
512	1	—	0.9154
	2	—	0.8556
	3	0.1149	0.8597
1024	1	—	0.9111
	2	—	0.8817
	3	0.1105	0.8883
2048	1	—	0.9272
	2	—	0.8948
	3	—	0.9197
